# Co-Designing a Digital Coach-Supported Parenting Program for Internalising Problems in Autistic Children

**DOI:** 10.3390/ejihpe16050071

**Published:** 2026-05-21

**Authors:** Olivia Bruce, Wan H. Sim, Aspasia Stacey Rabba, Anthony F. Jorm, Elizabeth Nicolaou, Ling Wu, Marie B. H. Yap

**Affiliations:** 1 School of Psychological Sciences, Turner Institute for Brain and Mental Health, Monash University, Melbourne 3800, Australia; 2 School of Educational Psychology and Counselling, Faculty of Education, Monash University, Melbourne 3800, Australia; 3 Melbourne School of Population and Global Health, University of Melbourne, Melbourne 3010, Australia; 4 Faculty of Information Technology, Monash University, Melbourne 3800, Australia

**Keywords:** co-design, internalising disorders, autism, children, parenting, parenting programs

## Abstract

Depression and clinical anxiety (also known as ‘internalising disorders’) are commonly experienced by autistic children. Parents play an important role in reducing their child’s risk of developing internalising disorders, and existing technology-assisted parenting programs have shown promise in empowering parents in this role. Yet, existing interventions do not currently meet the unique needs of parents of autistic children. This study aimed to co-design adaptations to an existing technology-assisted parenting program (*Partners in Parenting Kids*) to enhance its relevance and acceptability for parents of school-aged autistic children. An iterative two-phase co-design study was conducted with parents of autistic children (*n* = 5) and service providers (*n* = 5). In Phase 1, semi-structured interviews explored participant experiences and needs in the context of parenting support, as well as perspectives on parenting programs. In Phase 2, eight co-design workshops were conducted with parents and service providers to build on the findings from Phase 1 and to collaboratively adapt the program content, delivery, and design features. Workshops involved participatory design activities to foster collaborative sharing of ideas and decision-making. Transcripts from both phases were analysed using reflexive thematic analysis. Themes identified in Phase 1 included: (1) Day-to-day challenges of parenting an autistic child; (2) Unique parent knowledge base and skill set; and (3) Desired qualities of parenting programs. Themes from Phase 2 of the study included: (1) Meaningful connections with others in the community; (2) Acceptance of autism; and (3) Diversity within the community. These themes are described in terms of their design implications for the resultant parenting program (*Partners in Parenting Kids-Autism*). The findings provide critical insights into desired qualities of parenting programs for parents of autistic children. Importantly, they also shed light on key design recommendations for future work focused on empowering parents to support their child’s mental health through interventions.

## 1. Introduction

Between 1 and 3% of young people in Australia and around the world are autistic (Australian Bureau of Statistics, 2024; Maenner, 2020; Zeidan et al., 2022). Autistic people have distinct ways of communicating and relating to others, as well as unique patterns of focus, interests, sensory processing, and behaviour (American Psychiatric Association, 2022). Autistic children often experience co-occurring mental health challenges at a higher rate than their non-autistic peers, with anxiety and depression (also known as *internalising disorders*) among the most common (DeFilippis, 2018; Pezzimenti et al., 2019; van Steensel et al., 2011; S. W. White et al., 2009; Zaboski & Storch, 2018). For autistic children, these co-occurring problems can have significant impacts on child and parent functioning (Kim et al., 2000) and have been identified as risk factors for decreased life satisfaction and greater social difficulties in adulthood (Gotham et al., 2015). In a recent review, comorbid internalising symptomatology was negatively related to quality of life in adulthood (Sáez-Suanes & Álvarez-Couto, 2022). Together, this evidence suggests that early internalising disorders may contribute to poorer long-term outcomes in autistic children, highlighting the importance of addressing these challenges in childhood.

Parents play an important role in supporting their child’s mental health (Melchior & van der Waerden, 2016; Yap & Jorm, 2015). Childhood is a critical time of social and emotional development, when children rely on their parents for guidance and support. For parents of autistic children specifically, whose children may experience more emotional and social challenges than non-autistic children, their role in addressing the child’s internalising problems may be even more pivotal. Cognitive behavioural therapy for anxiety in autistic children, when implemented with parental involvement, has been shown to have greater effect sizes in reducing children’s anxiety symptoms as compared to child-only interventions (Perihan et al., 2020). Further, a recent qualitative study suggests many parents of autistic children are already involved in their child’s mental health treatment in various capacities, including as a co-facilitator, such as supporting the implementation of therapy both in and out of the session, or as a complementary helper, such as scaffolding the child’s participation in the therapy (Chan et al., 2025). Thus, arming parents with effective strategies to maintain this supportive role outside of therapy serves as a promising avenue to further reduce autistic children’s internalising problems.

Existing parenting programs focused on addressing child internalising problems in the general population have been shown to be effective in improving parenting (Buchanan-Pascall et al., 2018; Sim et al., 2020; Yap et al., 2016), but have two key limitations for parents of autistic children. First, the majority of programs were not developed with autistic or neurodivergent children in mind. While little is known about the specific strategies parents tend to use, evidence indicates that parents of autistic children adjust their parenting to manage additional challenges such as challenging behaviours, anxiety sensory processing difficulties, and sleep disturbances (O’Nions et al., 2018; Schaaf et al., 2011; Williams et al., 2006). Second, parents of autistic children hold various additional roles in their parenting, which may not be acknowledged in existing universal parenting programs designed without autistic children in mind. On top of supporting their child’s mental health, parents have a central role in supporting their autistic child in the context of initiating and navigating the diagnostic/identification process (Makino et al., 2021), managing various therapies (Cheng et al., 2023), and supporting their specific educational and social developmental needs (Fernández Cerero et al., 2024; Oppenheim et al., 2025; Siller & Sigman, 2002). Thus, it is important in the development of any parenting program that the needs of parents of autistic children are appropriately considered alongside the multitude of roles that parents must navigate. For example, many parenting programs or parent training programs which have been specifically developed for parents of autistic children tend to focus on externalising or disruptive behaviours, rather than internalising problems (Bearss et al., 2013, 2015; Tellegen & Sanders, 2014; Tonge et al., 2014). While adapted cognitive behavioural therapy and parent-mediated approaches for autistic children have demonstrated efficacy in reducing internalising symptoms (e.g., Perihan et al., 2020), these are primarily child-focused interventions with parent involvement, rather than programs that focus primarily on equipping parents with strategies they can use to support their autistic child’s mental health, that address the unique challenges and roles of parents of autistic children. To the best of our knowledge, there are limited evidence-based parenting programs focused on internalising problems in autistic children specifically.

### 1.1. Program Development

To address these gaps in existing parenting programs, participatory research approaches offer a valuable framework for developing or adapting new programs that are relevant, feasible, and responsive to parents’ needs and challenges. A growing body of research highlights the numerous benefits of directly involving service users in the design process of services and programs (Bate & Robert, 2006; Sanders & Stappers, 2008; Slattery et al., 2020) and specifically in the area of youth mental health (Chinsen et al., 2025). Further, the use of participatory approaches has been integrated in both health service and research standards/guides (den Houting, 2021; Department of Health and Aged Care, 2024; National Commission on Safety and Quality in Healthcare, 2021), highlighting how participatory methods are essential to ensuring research is accessible for members of the autism community and reflects their needs and priorities. The present study utilised co-design, which involves active collaboration between stakeholders in the design process (Slattery et al., 2020; Vargas et al., 2022). UK Design Council’s Double Diamond framework (2019) proposes a four-phase process for co-design: Discover, Define, Develop, and Deliver. It describes the design process of engaging directly with community members to discover and define what the problem is, then develop and co-design the solution, and deliver this on a small scale and engage in iterative improvement. This allows insights from stakeholders to be directly incorporated into the program and shape its development. It also allows for the leveraging of existing work, so the co-design can be specifically focused on addressing the needs of parents of autistic children.

### 1.2. Partners in Parenting Kids Program

The Partners in Parenting Kids program (formerly Parenting Resilient Kids or PaRK, herein *PiP Kids*) may be an ideal candidate for adaptation, as it is an established evidence-based, individually tailored, digital universal preventive parenting program. The online program consists of a tailored feedback report and up to 12 modules, recommended based on responses on a self-assessment tool of parenting risk and protective factors for child depression and anxiety disorders (Sim et al., 2019). PiP Kids is positioned as a universal prevention program at Level 3 of the PiP multi-level framework (Yap et al., 2017), whereby additional program components such as individual parent coaching sessions can be added depending on parents’ existing competencies or level of parental self-efficacy, or to address clinical-level internalising symptoms, thereby extending into indicated prevention or early intervention (Level 4). PiP Kids has demonstrated efficacy in improving parenting behaviours related to child anxiety and depression, as shown in a randomised controlled trial (Sim et al., 2020), with higher levels of parental engagement in the program predicting higher levels of preventive parenting and parental psychosocial health-related quality of life, and lower levels of impairments in child health-related quality of life (Sim et al., 2022).

Co-designed adaptations of the adolescent version of the Level 4 PiP program have demonstrated efficacy in more indicated-prevention or early-intervention populations, supporting parents of adolescents experiencing mental health challenges and co-occurring school refusal (Smout et al., 2025) and suicidality (Cao et al., 2025). This adaptability makes the PiP Kids program an ideal candidate for a co-designed adaptation to address internalising problems in other specific populations, such as for parents of autistic children.

### 1.3. Aims and Scope of Present Study

The aim of the present study was to co-design an adaptation of an existing evidence-based, digital parenting program (PiP Kids) to reduce internalising problems in autistic children. We aimed to understand the lived experiences and perspectives of parents and service providers and use these insights in the co-design process, which took place in two iterative phases. Across both phases, three research questions (RQs) guided the study:

**RQ1**. *What challenges do parents of autistic children encounter when supporting the mental health of their autistic children?*
**RQ2**. *What are the key components that parents of autistic children and service providers perceive as essential in online parenting programs designed to support child mental health?*
**RQ3**. *How can the content and format of online parenting programs be adapted to meet the needs of parents of autistic children, to enhance their effectiveness and accessibility?*

In Phase 1, we conducted qualitative semi-structured interviews to support the Discover and Define stages of the Double Diamond Framework (UK Design Council, 2019) and explore the needs and experiences of participants, specifically focusing on the first and second RQs. In Phase 2, we conducted co-design workshops to target the Define and Develop phases by collaboratively adapting and refining the program content and design to develop prototypes for two program modules, specifically focusing on RQ3. The two phases of the study were conducted sequentially, with the second designed to inform and build upon the first in an iterative fashion. Here, these phases are presented to illustrate how the findings from Phase 1 informed the Phase 2 co-design process and program development of a new online, coach-supported parenting program for parents of autistic children, called *Partners in Parenting Kids-Autism (PiP Kids-Autism)*.

## 2. Co-Design Group

### 2.1. Participants

The present study engaged five parents of autistic young people (*parents*, herein) and five service providers (*providers*, herein) as participants in the co-design group, in partnership with the researchers. Participants were invited to participate in both Phase 1 (Interviews) and Phase 2 (Co-Design Workshops) which took place across a period of 20 months between March 2022 and October 2023. Participants were aware that the study would be directly contributing to the development/adaptation of the new parenting program. The methods used in this study were approved by the Monash University Human Ethics Committee (ID #30478) and all participants gave written informed consent.

Five parents (four mothers and one father) were purposively recruited from a directory of parents who had previously taken part in a randomised controlled trial (RCT) of the original PiP Kids program (Sim et al., 2020) and identified as having autistic child(ren), and from the community through social media advertisements. The sample included parents who identified themselves as neurodivergent, as well as those who did not. Recruitment from prior trial participants may have introduced a bias towards parents who were potentially more positively disposed towards digital parenting interventions. However, this sampling approach was intentionally used to ensure participants had relevant experience with the intervention content and format, enabling informed contributions to the co-design process which focused on these elements of the intervention. Inclusion of participants without exposure to the PiP Kids program broadened the sample. This small purposive sample of individuals with rich, relevant experience was consistent with exploratory qualitative design, allowing for in-depth discussion and engagement in the iterative co-design process (Malterud et al., 2016).

Inclusion criteria required parents to either have an autistic child who is currently attending a mainstream primary school or an older autistic child who has finished mainstream or special school in the last two years. Parents of children who did not yet communicate by speaking, had significant motor or sensory impairments, or required acute treatment or intensive intervention (e.g., had behaviours that frequently result in physical injuries to self or others) were not included in the study. This exclusion was due to the first iteration of PiP Kids-Autism program being planned to support a broader group of parents facing less complex challenges, ensuring that the initial program could be appropriately tailored before addressing more intensive support needs. Parent participants were reimbursed AUD$35 per hour of participation in the study. See Table 1 for Parent participant demographics.

Five providers were recruited through the authors’ professional networks, advertisements on social media, and community groups in Australia. Inclusion criteria required providers to have five or more years of experience working with an autistic child or adolescent to participate in the study. Providers were reimbursed AUD$35 for their time spent participating in the study. See Table 2 for Provider participant demographics.

### 2.2. Researchers

Two female members of the research group were involved in the co-design group (authors WHS and OB). Author WHS (PhD, Research Fellow and Educational & Developmental Psychologist) served as the interviewer and workshop facilitator. As the lead researcher, WHS approached the study from a dual positionality perspective: as someone with familial connections to autism and as a health professional with extensive experience working directly with families of autistic children. Although four of the five parent participants had prior involvement in the RCT where author WHS was one of the researchers, there was no direct contact with these parents until invitations to participate in the present study were extended to them. Through her professional networks, author WHS was professionally known to three of the five providers. This pre-existing relationship was disclosed and carefully considered to prevent undue influence on participation. A second researcher, author OB (BSc(Hons), Clinical Psychology PhD Candidate and Provisional Psychologist) was involved in the study as a workshop co-facilitator and coder. Author OB also had a dual positionality perspective, with prior clinical and research experience working with neurodivergent young people and their parents, as well as a personal connection to autism. Author OB did not have a relationship with any of the participants prior to the present study. Both researchers engaged in ongoing reflexivity to remain aware of and manage the influence of both personal and professional experiences, as well as acknowledging how these shaped the research context and findings.

## 3. Phase 1: Interviews

The first phase of the study aimed to better understand participants’ perspectives on parenting an autistic child, with a focus on parenting to support child mental health, and the role and value of parenting programs. This phase involved one-on-one semi-structured interviews. Interviews were grounded in a phenomenological methodological orientation (Paley, 2025; van Manen, 2016), as the primary aim of this phase was to gain a deep understanding of participants’ lived experiences and perspectives regarding technology-assisted parenting programs in order to identify key adaptations to be co-designed. This approach allowed for parents and providers’ experiences to be foregrounded in the initial program conceptualisation, design, and adaptation, and served as an introductory dialogue to build rapport and a shared understanding with participants.

### 3.1. Methods

All ten participants participated in the interview phase. Semi-structured interviews were conducted using a pre-determined interview schedule by author WHS, who took written field notes during the interview. All interviews were conducted on Zoom©, and were audio- or video-recorded with participant consent. Interviews were first transcribed verbatim by a trusted third-party software program, Descript (Descript, see https://www.descript.com). To support rigour, transcripts were then manually checked and edited to ensure accuracy, but were not reviewed by participants. While member checking can enhance credibility, this approach was considered appropriate given the iterative, co-design nature of the study, where participants contributed to ideas across sessions after the interview, and to minimise participant burden.

Interviews with parent participants included two parts. The first half of the interview was focused on understanding the parent’s lived experience in parenting their autistic child, in particular managing their daily joys and challenges, and supporting their mental health on a daily basis. The second half of the interview was focused on understanding the parent’s views and experiences with using technology to access parenting support, and online parenting programs. Parents were asked a number of structured questions, with follow-up prompts to further understand their perspectives (see Supplementary Materials for full list of interview questions). Interviews with parent participants were between 65 and 131 min in duration (*M* = 88).

Interviews with providers were split into four parts. The first and second parts of the interview were focused on understanding the provider’s experiences supporting autistic children and their families and perspectives on the needs of parents of autistic children, with a particular focus on parenting. The third part of the interview focused on the provider’s perspectives on online parenting programs/information. The final part of the interview further explored service provider’s perspectives of indicators of progress and outcomes in parents and autistic children (see Supplementary Materials for full list of interview questions). Interviews with service providers were between 52 and 58 min in duration (*M* = 55).

### 3.2. Analysis

Interview transcripts from both participant groups were analysed using Reflexive Thematic Analysis, chosen as it allowed for a rich exploration of participants’ experiences while also acknowledging the active role of the researcher in interpreting the data (Braun et al., 2022). The analytic process involved a single coder (author OB), who first engaged in familiarisation with the data through reading of the transcripts and then inductively completed initial coding using NVivo 14 software (Lumivero, 2023). The author kept personal reflexive notes during the coding process. Groups of codes were used to generate preliminary themes, which were reviewed by the authorship group in sense-checking meetings. Themes were refined, named, and described, and finalised using illustrative quotes, reported using the Consolidated criteria for Reporting Qualitative research checklist (Tong et al., 2007), see Supplementary Materials. Throughout the analytic process, the research team (authors OB, WHS, and MBHY) met regularly and engaged in ongoing discussions to ensure depth and rigor, and all authors agreed on the final themes. Credibility and confirmability were supported by sense-checking meetings held with the research team and the inclusion of illustrative quotes. During sense-checking meetings, themes were discussed and disagreements resolved through collaborative discussion. The authors discussed each individual theme and the contributing codes to each theme. Divergent interpretations (e.g., divergences in the researchers’ initial responses to the interview content and the themes) were resolved by returning to the data and subsequent discussion, contributing to iterative refinement of themes. After each sense-checking meeting, a new version of the themes was documented on Google Documents until the final themes were agreed upon. Analytic decisions were considered to be made collaboratively; that is, the researchers had input on the themes at all stages of the analytic and write-up process.

Concepts of data saturation were not applied to our use of Reflexive Thematic Analysis (Braun & Clarke, 2021). The sample was considered to have sufficient information power due to the focused, specific study aim (co-design adaptations to a parenting program to reduce internalising problems in autistic children) and the inclusion of participants with highly relevant experience to the process of co-designing intervention content (parents with lived experience of supporting an autistic child, including those with prior exposure to the PiP Kids program, and providers who all had more than 5 years of relevant professional experience). The analytic approach (Reflexive Thematic Analysis) prioritised depth and shared meaning-making over breadth, further supporting adequacy with a smaller sample. Further, the iterative two-phase design of the study allowed for iterative idea generation, where participants could build upon ideas across the interview and co-design workshops. Together, these factors supported the adequacy of the sample for addressing the study aims. As our intention throughout the co-design process (both Phase 1 and 2) was not to explicitly compare the Parent and Provider groups, in the analysis we primarily aimed to identify shared perspectives. We recognise that the codes, and thus the interpretation of meaning within the transcripts, evolved throughout the analytic process and, thus, did not reach a fixed end-point.

### 3.3. Results

**Figure 1:**
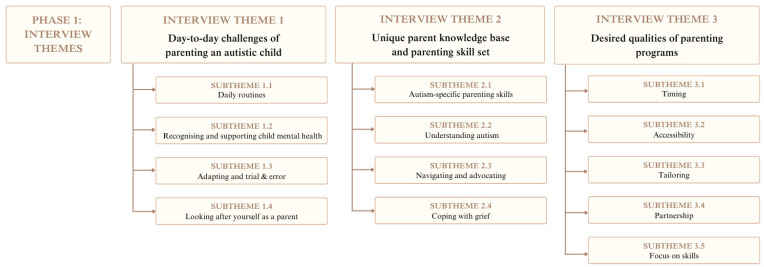
Themes identified from interviews (Phase 1).

From the interviews, three themes each with four or five subthemes were developed. These themes were: (1) Day-to-day challenges of parenting an autistic child; (2) Unique parent knowledge base and skill set; and (3) Desired qualities of parenting programs. For a summary of the themes and subthemes, see Figure 1.

#### 3.3.1. Interview Theme 1: Day-to-Day Challenges of Parenting an Autistic Child

Both participant groups identified a number of challenges of parenting an autistic child within their **daily routines** (Subtheme 1.1), such as supporting and guiding their child’s behaviour and sleep, managing their child’s sensory needs, and moving through their *“daily tasks”*, including going to school. Parent 2 reflected on encouraging their child to go to school, saying *“we have school refusal a lot as well. So it’s that coaxing of trying to get them to get ready for school and then do the school run”.* Managing sensory needs was also identified as a particular challenge. Parent 4 reflected on this complexity, saying *“the sensory system is such a complex thing… any sensory system in any combination can be out at any time, and I just wish sometimes that I had like a, if he’s doing these things try these things, without me having to go, now which sensory system’s out, what can we do here?”.* Parent participants also specifically identified that they struggle(d) with managing conflict at home (including between siblings), managing screen time, and negotiating with their child. Providers also highlighted the challenge parents face in identifying and differentiating between modifiable behaviour and autism-specific behaviour, saying *“[parents] have to learn how to differentiate, this presentation of behaviour that is like, because of the challenges or difficulties coming from the autism or this presentation of behaviour is actually a disciplinary issue” (Provider 5).*

Participants noted that parents may find it difficult to **recognise signs of mental illness** in their child (Subtheme 1.2), and that this was particularly challenging for parents of autistic children due to the other behaviours they have, such as difficulties with emotional regulation. One provider reflected *“I see a lot of co-occurrence of anxiety and a lot of the behaviours that present when, you know, when we unpack, is anxiety.” (Provider 3).* Providers noted that identifying mental health symptoms in the context of autism was an additional challenge for parents. As one provider explained, *“recognising the signs [of mental health conditions], because they may be nonverbal or they may not be able to express themselves verbally…it puts that other layer of complexity.” (Provider 1).*

Parents found **adaptation and trial and error** in their parenting to be challenging (Subtheme 1.3). This process occurred as parents adjusted their parenting strategies to meet the diverse needs of their children, particularly those parenting both autistic and non-autistic children. Parent 2 reflected on the differences between parenting their children, saying *“it’s like a completely different set of skills that you need to help one versus the other”*. Parents also found it challenging to adapt their parenting strategies when they stopped being effective, consider how and when they may share their child’s diagnosis with others in consideration of their child’s autonomy and privacy (acknowledging it is *“their story to tell”*), and learn about autism and develop their parenting skills. These may be important challenges to acknowledge and normalise within a parenting program. Parent 4 reflected that *“strategies wear out, but if you don’t know that as a parent. You’re like why is this working today or not working tomorrow? So you’re constantly having to come up with a new strategy and a new way of doing it”.*

Parents also identified they needed to adapt their parenting strategies to their individual context, particularly as their child grew. Although different developmental stages bring new challenges, Parent 5 identified enduring skills that she and her son learned in therapy, *“[to] have more like, I guess, tools in his tool belt with, with emotional regulation or just walking away from triggers, that kind of stuff. He’s come a long way. Puberty definitely have put a spin in things, making him a bit more reactive for the moment, but those skills that we’ve learned haven’t gone away”.*

Both participant groups emphasised the importance of parents **looking after themselves** (Subtheme 1.4), which was often neglected, and suggested this be foregrounded in parenting support. One parent worried her own mental health may affect her child, saying “*I find entering social situations, quite anxiety inducing. So I worry that I’m passing that on.” (Parent 4).* Providers identified several challenges parents face in reaching out for help. They acknowledged that many parents are already experiencing *“a high burden of care” (Provider 1)* in their parenting role, therefore self-care will often be de-prioritised. Provider 5 identified that often support will focus on the autistic child, rather than the parent. They reflected that *“not very often I will hear from the parents saying that actually, I also need some support. I also want to learn some parenting skills that help me to look after my child. Most of the time they will focus on the child or they are more eager to get support for the child because they don’t see that they are like really need that kind of support”*. Participants acknowledged the importance of shifting the focus from only offering support for the child, to a parenting program that centres the parent and their support needs, and promotes parent self-care.

#### 3.3.2. Interview Theme 2: Unique Parent Knowledge Base and Skill Set

Due to the unique challenges that their autistic children have, it was clear from the experiences of both participant groups that parents of autistic children develop a unique knowledge and skill-set. One parent reflected that *“as a parent, you’re having to deal with things that you probably wouldn’t have to deal with if your kid was typically developing. And it’s about trying to find the time to get those sorts of skills when you’re parenting three kids” (Parent 2).* This theme describes participants’ perspectives regarding these unique skills developed through the parenting journey, which included autism-specific parenting skills, developing knowledge about autism, skills in navigating and advocating, and coping with grief.

Both participant groups identified that out of necessity, parents of autistic children learned unique **autism-specific parenting skills** (Subtheme 2.1). These included learning how to manage aggressive behaviour. Provider 1 reflected on hearing parents *“describe situations where they’ve had to be hospitalised because of harm from their child….even then they find it really difficult to ask for help”.* Parents also identified learning how to do an increased amount of scaffolding and modelling for their child, particularly around social situations. For example, Parent 2 stated “*I feel like my entire life is about preparing social scripts”*. Parent (but not Provider) participants identified additional challenges, including managing developmental regressions in their child and learning to cope with their own worry about their child’s future and perceptions from others, such as *“worries and concerns will be how he’s perceived at school by teachers, how he’s perceived at school by peers” (Parent 5)* and *“my big concern for him … it’s acceptance in the community and in where they are and their acceptance of themselves within that community” (Parent 2).*

Increasing parental **understanding about autism** and neurodiversity (Subtheme 2.2) was a key area of development and adjustment for parents of autistic children, as identified by both participant groups. As Parent 3 said, *“I think the skills that are needed for that, I think parents who understand, who are willing to undertake some education and who are accepting of neurodiversity and are willing to put in the work to take their child to therapy, to learn the skills, to help so that they can help their child”.* Providers also highlighted the importance of parents having an increased and accurate understanding and knowledge about autism in order to support their child. Provider 5 reflected, *“I think for parents, they don’t have that kind of knowledge. And even after they do the assessment, most of the time in the debriefing they don’t have much time to understand what it is. So they just have a very brief concept. Some of them, they will just like Google and the information they get online may not be very accurate”.*

Both participant groups identified that an important and resource-consuming role of parents was **advocating for their children** within healthcare and education systems and learning how **to navigate these systems** themselves (Subtheme 2.3). Participants felt that a significant role of a parent of an autistic child is ensuring their child’s needs are met within the context of their healthcare, the National Disability Insurance Scheme (NDIS), school, and therapy, and this was identified as a contributor to parenting stress. Providers also identified learning about the different roles of clinicians and coordinating services as a unique skill parents develop, often out of necessity. This subtheme is poignantly illustrated by the following participants:

*“So the biggest stress with having a kid with autism is you are constantly fighting. You are constantly fighting. So you fight really, really hard to get their diagnosis and find someone who will listen to you. Then you fight really, really hard to find people that can help you, help them. And then you fight really, really hard to keep their diagnosis. And then you fight really, really hard with school to get them what they need and to get allowances made and to try and, and you fight like in social settings to try and get them some friends and stuff. And then you’re fighting the NDIS all the time. Like you have, I have to reprove that they’re autistic, like every time.” *

*(Parent 4)*

*“I’ve heard it time and time again from families that the whole process that, you know, Centrelink from Centrelink through to NDIS to the hospital system, it’s terribly demoralizing. And families are having to be the lynchpin or the central point of navigation and advocacy.”*

*(Provider 1)*

Navigating **grief** through the parenting journey was also identified by both groups, as parents learn to accept their child’s diagnosis, differences, strengths and limitations, and consolidate these with their expectations for their parenting journey (Subtheme 2.4). Both participant groups identified the process of acceptance of their child’s diagnosis as an important parenting journey which should be acknowledged in support for parents. From a Parent’s perspective, *“there is a grief process in that. Because you don’t go into having a kid thinking you’re gonna get a child with special needs, like, and autism is a double edged sword. It’s like, it’s a blessing in a lot of ways, but it’s hard in a lot of ways. And you can grieve different things…And I think that needs to be talked about more”* (Parent 4). From the Provider perspective, *“I would say for most of the parents that we have, you know, they go through that grief cycle. Right. You know, where they get the diagnosis and, and they have to come to terms with it. I mean, that in itself is quite traumatic for parents”* (Provider 4).

#### 3.3.3. Interview Theme 3: Desired Qualities of Parenting Programs

Turning from the experience of challenges and the development of unique parenting skills to perspectives of a more practical focus (specific components of parenting programs), both participant groups identified a number of desired design features of parenting support for parents of autistic children, including the timing of parenting programs, accessibility, individualised tailoring, partnership between parents and providers, and a focus on skills. While there is some inevitable overlap with the concepts described in Interview Theme 2, this theme focused on participant insights of a more practical nature, identifying directions for Phase 2 of the present study.

Key periods of time in the parent and child journey were identified as possible ideal **time periods** to provide focused and timely parenting support (Subtheme 3.1). These included key times where parenting support reduces; namely, (1) immediately after receiving a diagnosis and (2) after the child has received a diagnosis and the ‘early intervention’ period is over. Regarding the stage after diagnosis, Parent 2 reflected *“When people find out about a diagnosis, you wanna know all the information you can, but you get the information that’s relevant to that period. And so after you’ve done all that sort of early intervention, it feels like, everything after that is spot fires. Like you’re putting out spot fires for the issues at the moment and concerns rather than getting like a broad understanding of that time, because you’re still, you’ve gotta fit that in with everything else in your life I guess”.* Regarding the stage in the child’s development, one provider identified the primary school-age period as an important period to support parents. They reflected *“Those primary school years are a time when it can sometimes come unstuck…there’s a separation between what’s happening at home and what’s happening at school in those years quite often and often a disengagement from the early support team because they’ve transitioned into a different stage. And I think that sometimes that sets up a really unsupported time for families where they’re not directly communicating with what’s happening at school, they’re doing what they’re doing at home”* (Provider 2).

Both participant groups identified that it was important for parenting programs for parents of autistic children to be **accessible** to many parents within the autistic community (Subtheme 3.2). Participants identified needing to reach typically underserved populations and felt the program should be available to parents even if their child is not formally diagnosed as autistic; *“I think limiting the program to people whose kids have already received a diagnosis would be detrimental” (Parent 4).* Due to parents’ busy schedules and many parenting demands, participants felt that parenting programs should be relatively short and accessible to parents at times that work well for them or as a self-paced option. Provider 1 highlighted this variability, saying “*Any parenting program that’s developed needs to be short modules and it’s really accessible and really hits home with what’s the key message. what are the key learning almost straight away because, families just don’t have a whole lot of time”.*

In thinking about the different neurotypes and learning styles of parents, as well as practical accessibility, participants highlighted the need to have a diverse range of media types in a parenting program, rather than just written or spoken information. Similarly, Parent 5 identified this important diversity *“like, cause we all have different learning styles as well. Cause that’s what I’m doing at work at the moment, working with different learning styles. So not everyone is a reading person. Some people might need a video”.* Providers had similar ideas to improve accessibility *“or like audio books, if, you know, they have an audio book or podcast, as you said, so they can listen to it whilst they’re taking a walk or doing day to day things, rather than even finding five minutes in front of a computer, I think that’s challenging for lots of families” (Provider 1).*

Parent participants felt that often much of the parenting advice they find was not **tailored** to their specific child’s unique needs (Subtheme 3.3). Similarly, Providers identified that any parenting program should be tailored to the parent’s individual context, past experiences, existing skills, and child’s needs. Both groups clearly identified the need for individual tailoring of parenting support for parents of autistic children to the stage and needs of families. Providers affirmed the importance of taking an individualised approach and *“meeting parents where they are at”.* They reflected on their clinical work with parents, *“sometimes a lot of the work around those daily behaviours and things, it is very individualised and it would be, you know, having that conversation or kind of meeting each parent where they’re at as well” (Provider 3).* Similarly, another Provider reflected on their experience with supporting autistic children in an intervention context “*what we do has to be individualised for each child. you know, and we do tell parents, you know, this is not a cookie cutter approach” (Provider 4),* suggesting a tailored approach was strongly desired by participants.

Both participant groups identified the need for feedback and support with adapting and applying parenting skills, with both groups suggesting the program needed clinician or facilitator involvement and **partnership** to support this (Subtheme 3.4). Providers also identified the strength of this therapeutic relationship between clinician and parent as a key factor in the success of parenting support. Providers emphasised the importance of engaging parents as the experts in their own child and suggested a key feature of a parenting program is that it is run in partnership between clinicians and parents. One Provider reflected, “*I’m very much a firm believer that, you know, parents know their children better than anyone. They could have a PhD in them, really” (Provider 2).* Similarly, Parent 2 said *“yeah, because that was kind of like, you know you you’d go along and sometimes you’d sort of think, am I applying it right? Or things like that. And then just to sort of like get that little bit of feedback, that’s kind of like no, when we’ve tried this, my son’s done whatever. And then it’s just that little bit of tweaking”.*

A key valued feature of a parenting program was a **focus on skills** (Subtheme 3.5), rather than child behaviour or the child themselves, in order to support parents’ ability to adapt skills to their individual context. Participants acknowledged how different each autistic child was, further increasing the need to develop adaptable parenting skills. Another parent endorsed the role of parenting skill development as a key preventive strategy for their child’s mental health, *”I love the idea that it’s more of a preventative, you know, if you have the skills, then you can, you know, avoid him going down the path of having anxiety or depression” (Parent 3).* Providers emphasised the importance of focusing on equipping parents with skills through parenting programs, stating “*Has there been knowledge gained, but also have they been able to put that into practice?” (Provider 2)* and suggested identifying parent goals as a way to achieve this “*maybe [parents] could pre-identify, this is the thing I’m really struggling with, and then you could ask at the end, have you got a solution or have you seen an improvement in that particular issue that you’ve been tackling? But I think some measure of kind of knowledge translation might also be helpful” (Provider 2).* Provider 4 also felt skill development was critical for consolidating parent progress, as *“we don’t want them to be, you know, running these skills as we would in a therapy session, but we want them to generalise it and to just maintain, certain skills.”*

Participants felt a focus on skills allowed parents to learn strategies they could adapt for different situations and children. Parent 2 reflected on the value of adaptable skills for their family, *“so it was the fact that those kind of messages dealt with, like helped me with all my kids. Was something that we could set up across the whole family was really beneficial”.* Similarly, Provider 2 also identified this adaptability, *“the fact that it’s sort of structured and works through a series of, different, concepts and problems is helpful. And the fact that it covers things that they might not necessarily have experience yet, but helps them to give them a framework to navigate in the future.”* In thinking about the progress their child had made through the parenting skills they have learned, Parent 5 reflected on their progress, *“as we persist in doing different things, you know, whether it’s therapy or just doing different things as a family, you see how far he’s come and you realise that yep, it’s improving or whether it is his view of the world or, or how he’s reacting to different triggers.”*

## 4. Phase 2: Co-Design Workshops

The second phase of the study built on the findings from Phase 1, which served as a key framework to co-design and further develop specific elements of the program. This phase of the study involved iterative co-design workshops. These co-design workshops were aligned within a participatory design methodology approach and intended to involve parents and service providers as end users of the program, as per the Double Diamond framework (UK Design Council, 2019). The co-design workshops were designed to be a structured, collaborative space for participants to share their experience and view with the specific focus on extracting concrete design implications for the design and development of the program content.

### 4.1. Methods

The co-design workshops (*workshops*, herein) were conducted on Zoom© and were all facilitated by author WHS or OB. When required (if there were 2+ attendees), workshops were co-facilitated by author OB or another researcher, who was an honours student in Psychology. In each workshop, the ‘share screen’ functionality was used to collaborate on a shared digital brainstorming document (using Jamboard, a Google©-hosted software to create digital whiteboards) or review a module prototype (using Google Documents©). All workshops were audio- and visual-recorded and transcribed verbatim.

For each co-design workshop, all members of the co-design group were invited to attend. However, due to scheduling challenges, not all members of each participant group attended. In an effort to ensure the co-designed modules represented the co-design group’s perspectives, participants were offered the opportunity to contribute to module drafts asynchronously through Google Documents where relevant. Not all participants had capacity to do this, so it should be noted that outputs were likely shaped by the members of the co-design group who consistently attended the workshops to a greater degree.

In total, eight co-design workshops were conducted; five parent workshops between April 2022 and October 2023 which were between 91 and 121 min long (M = 104), and three service provider workshops between May and October 2022, which were between 58 and 92 min long (M = 78). A summary of the planned activities and attendees for each workshop can be found in Supplementary Materials.

The first two workshops focused on collaboratively identifying parenting challenges when supporting child mental health. We began with a structured Venn diagram discussion, where parents mapped challenges from their own perspective, challenges for their children, and areas where they overlapped. This visual exercise focused on creating a shared understanding of how parenting challenges are experienced differently, but also where they converge, such as in feeling disconnected or misunderstood, or experiencing a lack of acceptance. Following this, the ‘Three Wishes’ activity was used, in which parents imagined three wishes they would grant for their own child and for other autistic children and other parents. This reflective task elicited parents’ values and desired domains of life which parenting support might target, which they saw as the most meaningful. These structured activities provided a foundation for shared understanding and informed the foci for the later workshops.

**Figure 2:**
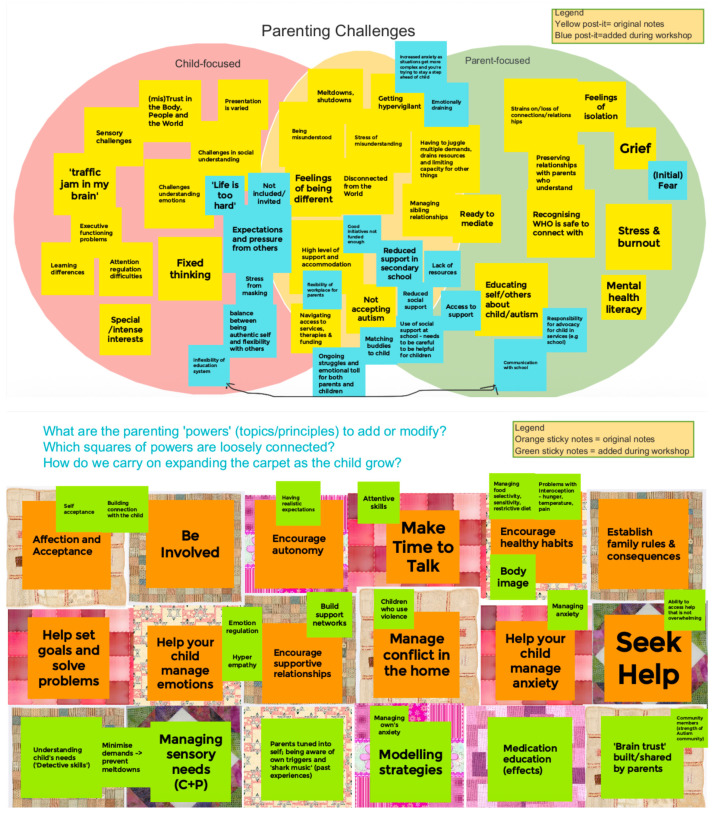
Examples of collaborative Jamboard slides from co-design workshops. (**Top**): Slide from opening discussion about parenting challenges, workshop 2 (Parent). (**Bottom**): Slide from ‘magic carpet’ activity, workshop 3 (Parent).

The next four workshops used interactive activities to deepen the exploration of parenting needs and supports. The first Magic Carpet activity involved a collaborative brainstorm where participants and facilitators built a symbolic ‘carpet’ made up of a mosaic of different ‘patches’, each representing a different element of parenting. Although originally designed for children (Clark, 2004), this Mosaic approach has been adapted for online delivery, providing a collaborative participatory method to listen to young people and adults’ stories and incorporate ideas (Clark, 2010). To begin with, the patches initially included the original PiP Kids parenting principles and the co-design group collaboratively contributed additional patches to capture more specific or nuanced aspects of parenting drawn from their lived experience (e.g., managing sensory needs, using ‘detective skills’ to understand the child’s needs), thereby identifying key domains of support that were most meaningful and practical. Complementing this, the Towers, Winds, and Storms activity provided a structured brainstorming exercise in which parents identified particularly challenging situations (the ‘storms’), contributing factors (‘winds’), and strengths and supports (‘towers’), that help them to navigate the parenting journey on the Magic Carpet with their child. Together, these metaphors encouraged reflection on both challenges and supports for parenting an autistic child. For all workshop activities, the facilitator and co-facilitator recorded ideas on the shared Jamboard page using sticky notes (see Figure 2 for examples).

In workshops three, four, and six conducted with providers, an additional Case Vignette of a child experiencing difficulties at school was introduced. Vignettes were used to support consolidation of discussions in real-life scenarios (Skilling & Stylianides, 2020) and help participants to feel comfortable discussing sensitive topics (Barter & Renold, 2000). This Vignette prompted discussion about parenting strategies that providers considered helpful for supporting parents in understanding the child’s challenges and responding constructively, offering insight into the types of supports that may be beneficial for parents. The Vignette was also used to discuss the role of the child’s school and partnerships between parents and their child’s school relevant to a parent caring for a child experiencing difficulties at school and mental health problems. This allowed for rich insights into the providers’ perspectives on the relationships between parents and other professionals supporting their child.

The final two workshops shifted to a hands-on review of program prototypes. For these workshops, two new drafted online modules were discussed, one new module initially called ‘Minding the self and finding your tribe’ and another module called ‘Autism: The fundamentals’. These modules represented concepts raised by the co-design group, which were not already covered in the PiP Kids program. Ahead of each scheduled workshop, the draft modules were shared in Google Document form with parents who were invited to review and provide written feedback through the ‘comments’ feature. During each workshop, facilitators guided a structured discussion about parents’ responses to the module, directly made suggested wording changes into the Google Document, and discussed the key ideas. We prioritised changes directly related to the ideas captured in the earlier Magic Carpet exercise, and discussions from previous workshops about common parenting challenges, language use throughout the program, and the framing of the strategies in the module, so the eventual modules stayed grounded in lived experience.

### 4.2. Analysis

Workshops were transcribed using the same process described in Section 3.2. Transcripts from both series of workshops were analysed using Reflexive Thematic Analysis, of which the general steps are described above (Braun et al., 2022). The note-taking products collaborated on during the workshops (e.g., the sticky notes contained on the Jamboard pages) were used to facilitate and visually organise discussion, rather than serve as standalone formal data sources and output of the workshop. The ideas captured in these artefacts were co-constructed through verbal interaction, and therefore represented within the workshop transcripts, which were formally analysed. To support analytic rigour, these artefacts were reviewed during the transcription process to corroborate interpretations; however, these were not independently coded, as this would have duplicated the transcript data.

Author OB again served as the sole coder, engaging with authors MBHY and WHS through sense-checking meetings and discussions. Because the workshops served to build on the findings identified in Phase 1, the workshops were analysed using a primarily ‘top-down approach’ (Braun et al., 2022; Pearson et al., 2025). That is, the workshops were primarily deductively coded using the same codes identified in Phase 1 and, in the cases of new meaning not represented in the Phase 1 coding, new codes uniquely identified from the workshops were added inductively. New codes were iteratively reviewed and treated with equal analytic weight as pre-existing codes identified in Phase 1 in the development of themes. This shift in coding approach reflected the sequential nature of the co-design process, moving from exploration (Phase 1) to application and design of concepts (Phase 2). This combination of processes was underpinned by pragmatism, and the iterative nature of the findings.

Codes (including both new codes identified in Phase 2 and codes identified in Phase 1 that were used to deductively code) were grouped to identify themes. Themes were identified with consideration to the overarching concepts generated during the co-design workshops, with codes iteratively organised first into preliminary categories, then into higher-order themes. Attention was given to ensure that themes represented the concepts most central to informing the intervention design. Further, these themes were developed to avoid redundancy with themes identified in Round 1. Therefore, codes which were only identified in Phase 1 and not elaborated or extended in the workshops were not carried forward into theme development for Phase 2.

For each participant group, the workshops were coded in the chronological order in which they occurred, in order to capture the generation of design ideas and insights as they evolved. Themes were reviewed by the research group in sense-checking meetings following the same structure as those described in Section 3.2. Themes were refined, named, and described, and finalised using illustrative quotes. The final three overarching themes were derived from this process, reflecting clusters of codes most consistently represented across the workshop data. Illustrative quotes are used to exemplify the themes.

### 4.3. Results

Across the eight workshops, three overarching themes relevant to program development were developed. These themes were (1) Meaningful connections with others in the community; (2) Acceptance of autism; and (3) Diversity within the community. Each of the themes elicited three design implications for the co-designed parenting program.

#### 4.3.1. Workshop Theme 1: Meaningful Connections with Others in the Community

Participants felt that meaningful connections to others in the community was important, identifying two key opportunities for the parenting program to equip parents with parenting strategies: building relationships with others, and parent relationships with the wider community.

First, regarding opportunities to support parents in building relationships with others, participants identified that parents of autistic children often needed to support their child to form meaningful relationships with others, including other children. Parent 4 identified their child’s relationship with other autistic children was an important part of their development, saying *“we’ve found that as our kids have got older they’ve actually been that support to their friends who are on the spectrum or to kids who need help…cause they understand what they’re going through”.* Participants also identified that parents themselves need to forge their own meaningful relationships with others, serving as a way for them to ‘find their tribe’ and build their support network. Parent participants reported it was important to form connections and relationships with other parents, in particular *“preserving those relationships with other parents that do understand” (Parent 3)* and *“feeling welcomed and accepted, but also not feeling like you’ve gotta explain everything or have excuses for everything” (Parent 2)*.

Second, participants also recognised an opportunity for the program to help parents regarding their relationship to their wider community through providing important resources. Parents felt that education of others in the community (e.g., grandparents, family members, teachers) about autism was necessary and that the information about autism contained in the program could also be made available *“to the wider public”.* Providers, on the other hand, highlighted the relationship between parents and their child’s service providers, sharing that it would be important to consider when and how they may be involved. Providers also emphasised the importance of working in partnership with the child’s school, particularly if the child was experiencing school avoidance related to emotional distress. To this end, participants felt that the support provided to parents for relationship-building should not be limited to the child’s relationships but also extend to parents’ relationships with other important people in their community.

Workshop Theme 1 led to three key design implications for the program:

*Design Implication A:* Design program materials to help parents to support their child in forming meaningful connections with others in the community, including their service providers.

*Design Implication B:* Design program materials to support parents to form and maintain connections with others in the community.

*Design Implication C:* Ensure program materials are developed to also serve a dual purpose as an educational resource which can be suitable for community members such as grandparents, family members, teachers, school staff, and service providers.

#### 4.3.2. Workshop Theme 2: Acceptance of Autism

During the workshops, the co-design group communicated their ideas within the lens of a neurodiversity-affirming paradigm. Specifically, participants expressed discomfort with deficit-focused language, preferring to use language such as *“differences”*, which acknowledges that *“everybody’s diverse”.* Building upon this understanding within the workshop environment, a neurodiversity-affirming paradigm was seen as paramount for the resultant parenting program. For example, Parent 3 said “*I think there’s a lot of pride within the neurodivergent community, and there’s certainly many strengths to being on the spectrum. So I’m sure your program is hopefully talking about neurodivergence as it’s something that’s blame free and shame free”*, and Provider 1 shared that they hoped parents were able to be *“proud of, you know, accepting who they are, young people…and they have the ability to educate their peers a little bit about what they need and what they want and how they like communicate”.*

Parents and providers felt that it was critical that parents of autistic children express towards their child *“acceptance for who they are” (Provider 1)* and that children are supported to be their *“authentic selves” (Parent 3).* Participants felt it was important to support parents to come to an acceptance of their child’s autism and autistic identity, allowing their child the freedom to be their authentic self and embrace their special interests. This acceptance would allow parents to set realistic expectations for their children and work towards achievable goals of improving their mental health without trying to change who they are. Parents also felt that their children were *“bombarded by negativity all the time”* *(Parent 2)* so participants valued giving other parents hope for the future by identifying their child’s strengths. Parent 1 reflected, *“we’ve got children who can be such a blessing to the community. And yet they get put down, and how much better the world would be if our children were allowed to flourish and be themselves”.* Parent 3 reflected on their hopes for their daughter, saying *“I want her to be that authentic self. I don’t want her to be the person that she thinks other people thinks she needs to be”.*

Workshop Theme 2 led to three key design implications for the program:

*Design Implication D:* Position the parenting program using a neurodiversity-affirming paradigm (i.e., viewing autism as a brain difference, rather than as a pathology to be fixed).

*Design Implication E:* Develop program materials to help parents set realistic expectations for their child.

*Design Implication F:* Develop program materials to help parents identify and foster their child’s strengths.

#### 4.3.3. Workshop Theme 3: Diversity Within the Community

Participants valued considering the diversity within families and within the autism community, highlighting families are *“from all different backgrounds” (Provider 1).* Parents identified that they often need to adapt their strategies in consideration of the differing needs of all children within the family, which needed to be accounted for in a parenting program. Parent 4 identified this need to consider siblings, saying *“disconnectedness is a word that comes to mind because they get disconnected from the world around them and you get disconnected from the world around them and their siblings get disconnected in various ways, from things too, depending on how severe they are”*. Participants valued diverse social, cultural, autistic, and disability representation in the media and vignettes, to ensure all parents felt included. As a concrete example, participants highlighted the need to consider First Nations parents, to ensure program materials were relevant and appropriate. Parents also acknowledged the limited diversity in the co-design group itself, noting the involvement of only one Father, and emphasised the importance of ensuring the program was inclusive of all parent roles.

Participants also felt it was important for parents to reflect about the neurodiversity within their family, and to be supported in acknowledging and accepting their own neurotype/neurodivergence, and for parenting programs to acknowledge diverse parent communication, sensory preferences, and needs, in particular autistic parents. As Parent 3 reflected, *“I think [it’s] so important, like if the parents have the education and the awareness to frame what their children or family members are, that they’re living in a neurodiverse framing”*. Regarding sensory preferences in particular, participants identified that other parents might struggle with sensory under- or over-stimulation themselves, so may benefit from specific sensory strategies. Parent 4 shared their own experience of this, reflecting *“I’ve had to start saying things like ‘can you not touch me right now’. Cause my son is like very physical touch oriented, and it’s like, I really can’t have you touch me right now, my sensory system is overwhelmed”.*

Workshop Theme 3 led to three key design implications for the program:

*Design Implication G:* Develop program materials to help parents to identify and support the needs of all children in their family.

*Design Implication H:* Develop program materials to support parent reflection on their own needs, communication style, preferences, and neurodiversity.

*Design Implication I:* Develop program materials to ensure that the media, images, vignettes, and examples reflect a range of diverse individuals.

## 5. Integrated Findings and Program Prototype

**Figure 3:**
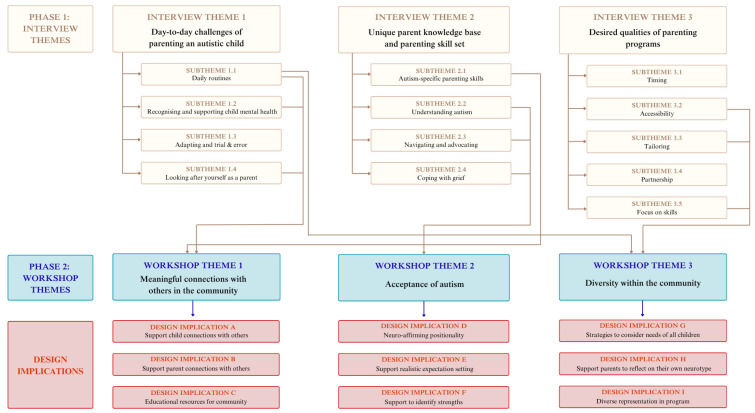
Themes identified from Phase 1 and Phase 2 and relevant design implications.

The themes from both phases of the study are presented in Figure 3, displaying how the foundational key themes of Phase 1 formed a basis for the themes from Phase 2, which informed the nine key program design implications.

### 5.1. Program Development Framework

Given the iterative nature of the co-design process, the development of the program content was guided by the themes and subthemes from both Phase 1 and 2, with the design implications iteratively developed and used to inform the intervention development process. Selected examples (presented in Section 5.2) illustrate how participant contributions shaped module content. The overall adaptation and development of the *Partners in Parenting Kids-Autism (PiP Kids-Autism)* program involved the following decision-making process.

First, adaptation decisions were anchored by the Parenting Guidelines underpinning the PiP Kids intervention (Parenting Strategies Program, 2014) which were developed through a prior systematic review (Yap & Jorm, 2015) and Delphi study of international expert consensus (Yap et al., 2015). These guidelines represented the non-negotiable ‘active ingredients’ or core components of the intervention and served as the primary decision-making framework to ensure fidelity to evidence-based parenting principles. All adaptations were therefore evaluated for their consistency with these core components (Escoffery et al., 2019).

Within this framework, adaptations were made to address findings from the present study by: (1) contextualising recommended parenting strategies for autistic children (e.g., incorporating guidance on adjusting expectations identified by Design Implication E around communication and sensory sensitivities); (2) explicitly acknowledging the need for parents to flexibly tailor the application of evidence-based principles (e.g., when developing and implementing family rules; incorporating Design Implications E and G); and (3) providing concrete examples of activities and strategies that are developmentally appropriate and acceptable for autistic children (e.g., using special interests within positive interactions and communication styles preferred by the child; incorporating Design Implication F).

Adaptations were considered across all modules, including the addition of new material (e.g., additional strategies on connecting with the autistic community and autistic role models), adapting existing module content to enhance relevance (e.g., replacing ‘talk with your child’ with ‘connect with your child’) and, where necessary, removal of non-core content deemed less applicable to parents of autistic children (e.g., removing examples of passive, assertive, or aggressive communication styles which did not consider autistic communication). The authors considered the extent of the adaptations required to address the design implication, adding new module pages as appropriate. Where it was not possible to address the design implication through the adaptation of existing content and the addition of module pages (e.g., there was too much new content to include), a new module was developed.

The findings of the present study informed the decision to develop four new modules; namely, ‘Autism: The fundamentals’, ‘Look after yourself’, ‘Guide your child’s behaviours’, and ‘Support your child’s sensory needs’. The development of new modules followed the same rigorous approach used to develop the original PiP Kids program (Fernando et al., 2018; Yap et al., 2017), drawing on the highest-available evidence and clinical guidance to ensure alignment with the overarching PiP Kids modules. For example, the ‘Look after yourself’ module was informed by evidence on caregiver mental health and self-compassion approaches (e.g., Catalano et al., 2018; Morgan et al., 2016; Kienhuis & Avdagic, 2021), integrating strategies shown to reduce parental stress with the lived experience insights from the co-design process.

After the first drafting process was complete, a working group comprising all authors iteratively reviewed the draft of each individual module. The working group met via Zoom for over 38 h in total, reviewing all adapted, added, or removed content in accordance with the framework above. The group collaboratively made design decisions based on the findings of the present study, participant preferences, and the overall approach and evidence-base of the PiP Kids program. Drafts of the modules were iteratively reviewed and re-reviewed until all authors agreed on the final intervention content.

Some themes and design implications were addressed throughout the entire program (e.g., Subthemes 3.1–3.5 and Design Implications D, F, and I). For example, timing was addressed through the program target age range (primary-school aged autistic children), focus on skills was addressed through the program’s focus on strategies and the addition of a coaching component, individual tailoring was addressed through the program’s personalised parenting feedback report and module recommendations and, finally, diverse representation through the addition of images portraying families from a diverse range of backgrounds. See Table 3 for a summary of all of the fifteen modules in the resultant program and the key adaptations which were made for the PiP Kids-Autism program.

### 5.2. Examples of Application of the Program Development Framework

Here, two examples of how the findings were applied in the development process are presented. These examples relate to two of the program modules, ‘Look after yourself’ module and ‘Seek help’.

#### 5.2.1. Example 1: Meaningful Connections in the Look After Yourself Module

After the co-design workshops, it became clear in sense-checking discussions between authors OB, WHS, ASR, AFJ, and MBHY that the strategies related to parental self-care and parents forming meaningful relationships with others both contributed to the overarching concept of parents looking after themselves. Therefore, the prototyped new module developed for the PiP Kids-Autism program was renamed from ‘Minding the self and finding your tribe’ to ‘Look after yourself’. Parents consistently emphasised the focus on these concepts in both the interviews and workshops; for example, Parent 3 stated *“my wish for other parents is that they can find their tribe”* and Parent 1 stated *“self-care I think’s super important, but explaining what it means, it’s not just your health, it’s your wellbeing”.*

**Figure 4:**
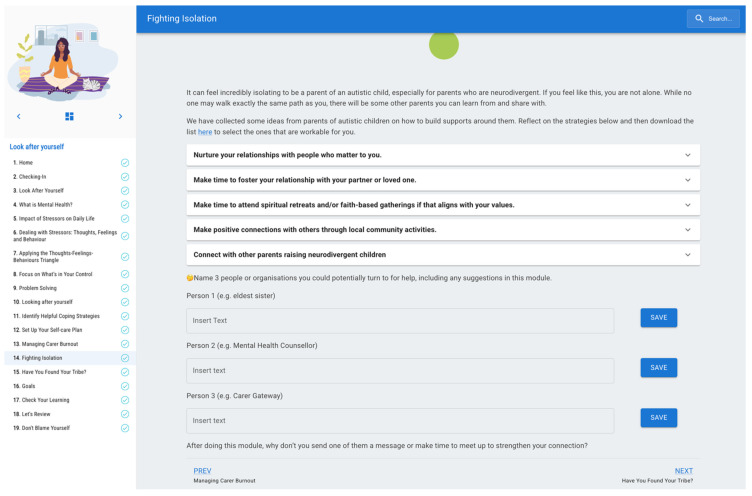
Sample page from module ‘Look after yourself’ in the Partners in Parenting Kids-Autism program.

Therefore, in this module, Design Implication B was incorporated to support parents to build meaningful relationships with others. This was added to content informed by Subthemes 1.4 and 2.3, where parents learn strategies to nurture their relationships with others in their community, identify connections with people who align with their values and identify people or organisations they may connect with for support when needed (see Figure 4 for a sample).

#### 5.2.2. Example 2: Support Bus Concept in the Seek Help Module

**Figure 5:**
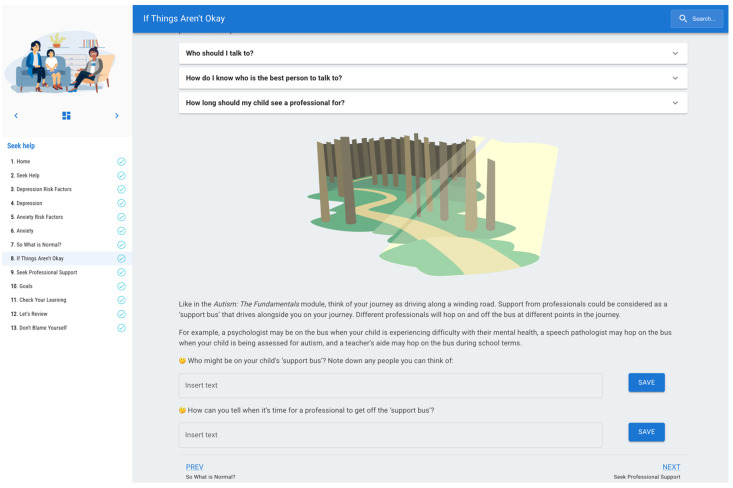
Sample page from module ‘Seek help’ in the Partners in Parenting Kids-Autism program.

Design Implication A, for example, was incorporated into a different program module. The concept of the ‘support bus’ was suggested by Providers in the workshops as a way of considering when to involve different service providers in a parent and child’s journey. Provider 4 brainstormed this concept, saying parents on the bus could *“at certain stops, you know, hop off the bus because they need that additional support or information…so it’s almost like a scheduled stop, almost like when you think about school for children, you know, it’s there, you know, it, the bus is gonna stop because that is part of the development of children’s lives. So parents can choose, okay yes, I need some more help with this. Or I don’t know about this. I don’t know enough about this. I’m gonna hop off and I’m gonna find out some more information here”.* The inclusion of this concept was also supported by Interview Subtheme 3.4 (Partnership) and Workshop Theme 1 (Meaningful connections), where participants highlighted the importance of partnering with service providers and clinicians to implement parenting strategies and work through challenges that arise. To address these findings, the ‘support bus’ concept was integrated into an existing module called *Seek help*, which helps parents to identify the symptoms of depression and clinical anxiety, and know when seeking further support from a health professional is necessary. Informed by the design implications from the present study, where the co-design group highlighted the importance of the child’s and parent’s connections and relationships with their child’s service providers (i.e., Design Implications A and B), the ‘support bus’ metaphor was integrated into this module. This aims to support parents through psychoeducation and guided reflection questions to identify which service providers the child might have a relationship with and consider when they might need to be involved or ‘hop off’ the ‘support bus’ (see Figure 5).

## 6. Discussion

The present study aimed to co-design adaptations to an existing evidence-based, digital parenting program to reduce internalising problems in autistic children, across two iterative co-design phases.

Phase 1 identified that despite the day-to-day and autism-specific challenges that parents of autistic children face, including understanding autism and looking after themselves as a parent, they have a number of important strengths and develop unique skills and knowledge. By better understanding these challenges, Phase 1 addressed RQ1: ‘What challenges do parents of autistic children encounter when supporting the mental health of their autistic children?’ The findings from Phase 1 also suggested that parents and providers value parenting programs that are accessible, available at important periods of time in their journey, tailored, focused on skills, and provide facilitator partnership and guidance. By identifying these elements of parenting programs, these insights addressed RQ2: ‘What are the key components that parents of autistic children and service providers perceive as essential in online parenting programs designed to support child mental health?’.

Building on these insights, Phase 2 aimed to address RQ3: ‘How can the content and format of online parenting programs be adapted to meet the needs of parents of autistic children, to enhance their effectiveness and accessibility?’ by co-designing program content. The findings from this phase identified three key themes of Meaningful connections with others in the community, Acceptance of autism, and Diversity within the community. These themes gave rise to key design implications, which offer insights into the preferences of parents and providers for parenting programs. These findings were co-designed into the resultant *PiP Kids-Autism* parenting program which consisted of 15 modules, and also offer insights for clinical services or programs focused on empowering parents of autistic children.

A finding of our study is the participants’ preference for a parenting program to focus on skills, rather than directly on child behaviour or mental health symptoms. Our findings suggest that parents and providers who formed the co-design group valued parenting programs that equip parents with strategies they could adapt to their context and apply across different children and situations. The challenges identified by the present study, such as identifying mental health symptoms in the context of autism, may be suitable as a potential skill that parents are supported to learn or develop through a parenting program. Looking forward to intervention development specifically, these insights suggest *skill development* should be considered a central component of programs for parents of autistic children. Future research focused on developing intervention materials for parents of autistic children may consider these findings in the framing of intervention content.

Taken together, our findings underscore the importance of *relationships*, as parents and providers emphasised the value of two key types of relationships for parents of autistic children. The first type of relationship focuses on the partnership between parents and their service providers, both their child’s and their own. Our findings suggested that the co-design group desired and valued the involvement of someone to support parents to implement the parenting strategies they learn and to adapt them to their situation. This suggests that the co-design group identified that incorporating a dedicated coach into the PiP Kids-Autism program may help to strengthen parents’ confidence and ability to apply the parenting strategies, while also fostering a sense of partnership between the parent and their coach. In the existing literature, a number of existing interventions for parents of autistic children utilise a service provider or therapist to perform a coaching or assistance role (Cheng et al., 2023; Rogers et al., 2019; Salomone & Maurizio Arduino, 2017), suggesting this may be a potentially feasible addition to the PiP Kids program in line with participants’ preferences as expressed in the present study. This also is aligned with the highest level of the PiP multi-level framework (Yap et al., 2017) as well as findings from existing coach-supported PiP+ programs (Fulgoni et al., 2019; Khor et al., 2022; Smout et al., 2025), which have identified the feasibility of coach involvement in parenting programs.

The second type of relationship pertains to the connections between parents of autistic children and their wider community. This includes building support networks among parents of autistic children and increasing awareness and understanding of autism in the community, encompassing grandparents, teachers, extended family members, and other parents. Providers also emphasised the role of parents’ relationship with their child’s school, highlighting the importance of considering school avoidance in autistic children who experience mental health challenges (Adams, 2022). To support this, it was suggested the PiP Kids-Autism program should contain resources which parents could then share with significant others in the child’s life. For example, parents valued resources offering practical guidance on communication strategies and managing sensory and social demands, which they can share with teachers and family members to enhance support for their autistic child. This may be particularly relevant for parents of children with higher support needs who require additional support in their community or school context, who were not represented in the present study. Further research is necessary to co-design resources to support parents in this area.

Participants also emphasised the importance of positioning programs/services to be strengths-based, and grounded in accepting children’s autistic identity (Workshop Theme 2—Acceptance of autism). It was important to parents and providers that the program supported parents to accept their child, identify their strengths, and better understand what autism means for them, as an essential aspect of empowering parents to support their child. Existing studies suggest strengths-based approaches are acceptable and effective in the context of child mental health services for young people (Fu et al., 2025; Turner & Mueller, 2025) and parenting programs (Waters & Sun, 2016). For interventions for autistic young people and their parents specifically, strengths-based approaches are positively regarded by parents (Shochet et al., 2019; J. White et al., 2025). While the present study did not aim to evaluate the acceptability or efficacy of the resultant program, this focus on strengths emphasised by the co-design group aligns with emerging evidence that suggests community preferences for services and research which affirm neurodivergent identities and involve community members (Pellicano et al., 2014; Roche et al., 2021). While there is a growing body of research defining neurodiversity-affirming or ‘neuro-affirming’ approaches (Flower et al., 2025; Izuno-Garcia et al., 2023), there is no consensus or gold standard for applying neurodiversity-affirming practice principles in interventions for autistic young people or their families. More research is required to more clearly integrate these approaches into parenting programs.

### Strengths and Limitations

The present study is strengthened by the use of co-design methodologies, which facilitated an authentic partnership between the research group and key stakeholders to co-design the PiP Kids-Autism program. The inclusion of both parent and provider perspectives allowed for partial triangulation of the unique insights from these key stakeholder groups, strengthening the depth of these findings and their impact on the intervention development. Although the use of digital technologies has inherent constraints (such as relying primarily on verbal discussion, reducing access to non-verbal cues such as body language and tone, and potential underrepresentation of individuals with limited access to digital technologies), the use of co-design activities conducted on Zoom allowed for the involvement of parents and providers from across Australia. We also note the limited reporting of sociodemographic variables of the participants in the present study, while intentionally limited to protect participant confidentiality, may impact the interpretability of the findings.

We acknowledge the sampling approach of the present study, which utilised the research team’s existing networks (Providers) and participants from a previous RCT (parents), has several limitations. While the familiarity of some members of the co-design group with the PiP Kids program or members of the research team allowed for the co-design process to be focused and contextually relevant, these pre-existing relationships may have influenced the perception of parenting programs and/or the interpretation of the findings. These relationships may have introduced social desirability biases, reduced willingness to express critical views, or a tendency to emphasise more favourable perspectives regarding intervention content. While we engaged several strategies to mitigate these influences, including having multiple facilitators, encouraging diverse perspectives during workshops, grounding analysis in verbatim transcripts, and engaging in reflexive discussions as a research group, the study findings should be interpreted in consideration of this limitation.

Further, a notable limitation of the study is its primary focus on parenting strategies for primary school-aged children within an English-speaking community. This specificity may limit the generalisability of the findings to parents from non-English-speaking backgrounds. Although the study’s small recruited sample size allowed for in-depth collaboration with participants, it also resulted in limited diversity within the co-design group, notably with respect to parents of children with higher support needs who did not meet inclusion criteria. Autism is heterogenous, and each autistic child has their own unique support and communication needs. Involving parents with a more diverse range of parenting experiences and engaging service providers with varied expertise in the co-design process may be a key area of development for future iterations of the PiP Kids-Autism program. This includes engaging parents of children with learning disorders or giftedness, as well as those caring for children who may not communicate verbally or have a co-occurring intellectual disability. Involving a broad spectrum of parents and service providers would enhance inclusivity and help to ensure that a program meets the complex and varied needs of the autistic community, further building upon this initial co-design effort.

## 7. Conclusions

The present study describes the process of co-designing a new digital coach-supported parenting program for autistic children with parents and providers. Our findings identified key themes and design implications for supporting parents of autistic children, which were integrated in the resultant program Partners in Parenting Kids-Autism. The program is being piloted in a single-armed pilot trial, which is prospectively registered with the Australia New Zealand Clinical Trials Registry (#ACTRN12624000394549). These findings provide insights into the desired qualities of parenting programs for this population, which are relevant for future intervention development efforts to empower parents of autistic children.

## Figures and Tables

**Table 1:** Participant demographics: Parents.

Parent	Parent Role	Number of Autistic Children (Total Number of Children)	Age of Autistic Child (ren)	Parent Previously Completed PiP Kids Program	Partner Previously Completed PiP Kids Program
1	Father	3 (4)	7, 12, 17	N	Y
2	Mother	2 (3)	12, 16	N	N
3	Mother	1 (2)	10	Y	N
4	Mother	2 (2)	12, 16	Y	N
5	Mother	1 (2)	13	N	N

**Table 2:** Participant demographics: Service Providers.

Service Provider	Gender	Professional Role	Years of Experience
1	Female	Health Service Manager	>5
2	Female	Paediatrician	>20
3	Female	Developmental Educator	>10
4	Female	Behaviour Therapist/Analyst	>10
5	Female	Psychologist	>5

**Table 3:** Summary of modules included in the Partners in Parenting Kids-Autism program and key adaptations made.

Existing or New Module	Module Name	Synopsis	Key Adaptations Made for PiP Kids-Autism	Relevant Phase 1 Theme and/or Phase 2 Design Implication
Existing	Show affection and acceptance	Highlights how parents can show their child physical affection and acceptance through words and actions	Content adapted and added to provide strategies to show affection in ways other than through verbal communication and consider how their child prefers to receive affection. Content added to support child’s understanding of affection and relationships.	Subtheme 1.3; 2.1 Design Implication A; E
Existing	Connect	Provides strategies for parents to develop a supportive relationship with their child	Content added to support parent reflection about differences in communication between parents and children. ‘Family talk time’ reframed to ‘family connect time’.	Subtheme 1.3; 2.1; 2.2 Design Implication A; H
Existing	Be involved and encourage autonomy	Shows parents how to stay involved and interested in their child’s life and helps parents to encourage increasing age-appropriate autonomy in their child’s life	Content adapted to introduce consideration of child’s support needs and ‘age-appropriate independence’ reframed to ‘developmentally appropriate independence’. Content added to include new skills and behaviour development.	Subtheme 2.1
Existing	Encourage supportive relationships	Provides strategies for parents to facilitate their child’s social skills development	Content adapted to provide extra consideration to social challenges. Content added to support parents to identify challenging social situations for their child, stranger safety tips, and develop social scaffolding skills such as social scripts and connect with autistic community and role models.	Design Implication A, B, E
Existing	Establish family rules and consequences	Provides strategies for parents to establish consistent and clear boundaries for child’s behaviours	Content added to support parents to identify when flexibility in rules and consequences as appropriate, such as when the child is experiencing a meltdown or in autistic burnout. Content added to support parents to pre-plan rules for different environments.	Subtheme 1.1; 1.3 Design Implication G
Existing	Encourage good health habits	Highlights how parents can cultivate good health habits related to diet, physical activity, sleep and screen use in their child	Content adapted to be more inclusive of common challenges in daily routines. Content added to support sleep in autistic children and identify appropriate physical activity activities.	Subtheme 1.1 Design Implication E
Existing	Manage conflict in the home	Addresses the need for adaptive conflict management between parents and between parent and child, and presents strategies to do these	Content added to support parents to manage conflict when child is in a meltdown or shutdown. Content added to support managing conflict between family members.	Design Implication A; G
Existing	Manage everyday emotions	Provides strategies for parents to assist their child manage their everyday anxiety	Content added to acknowledge the impact of alexithymia and help parents identify their child’s emotional experience through all forms of communication and expression. Content added about autistic children’s possible experience of negative self-talk.	Subtheme 1.2; 2.1
Existing	Manage tough emotions	Provides strategies for parents to help their child manage strong emotions	Content adapted to consider reasons why autistic children may be anxious and how autistic children may show anxiety. Content added to provide parents with strategies if their child cannot verbalise their anxiety.	Subtheme 1.2; 2.1
Existing	Support goal setting and problem solving	Shows parents how to support their child in developing problem-solving skills	Content added to help parents to identify common factors which influence goal setting and problem solving, such as executive functioning, motor skills, autistic burnout, difficulty with understanding other’s perspectives, and stress from the sensory environment. Content added to support parents in identifying challenges their autistic child might have at school and work collaboratively with their child’s school to support their school attendance and experience.	Subtheme 1.3; 2.2
Existing	Seek help	Addresses how clinical anxiety and depression can look like in children and what parents can do if their child is or becomes unwell	Content added about autism-specific clinical anxiety and depression risk factors and symptoms. Content added to support parents to find the right service provider, build a relationship, and know when they should be involved.	Design Implication A, B
New	Autism: The fundamentals	Provides information about autism, neurodivergence, and strategies for managing their energy as a parent	N/A	Subtheme 2.2; 2.3; 2.4 Design Implication C, D, F
New	Look after yourself	Provides information about relationships between Thoughts–Feelings–Behaviour, addresses the importance of self-care and meaningful connections with others and noticing the signs of carer fatigue or burnout, and presents strategies for parents to look after themselves	N/A	Subtheme 1.4; 2.3 Design Implication B
New	Guide your child’s behaviours	Addresses the function and factors impacting child behaviour and what parents can do to guide their child’s behaviour in everyday life, provides strategies for managing meltdowns and shutdowns	N/A	Subtheme 1.1; 2.1
New	Support your child’s sensory needs	Recognises the impact of sensory needs in daily life and the role parents can have to support this	N/A	Subtheme 1.1; 2.1

*Note:* Existing refers to an existing module in the PiP Kids program (Sim et al., 2020). New refers to a newly developed module based on the findings of the present study. Some module names of existing modules were reworded to align with the entire suite of PiP programs. Be involved and Encourage autonomy are two separate modules in the PiP Kids program and were combined into a single module in the PiP Kids-Autism program.

## Data Availability

The datasets presented in this article are not readily available due to the identifiable nature of the datasets. Requests to access the datasets should be directed to Professor Marie Yap at marie.yap@monash.edu.

## Supplementary Materials

The following supporting information can be downloaded at: https://www.mdpi.com/article/10.3390/ejihpe16050071/s1, Figure S1: Consolidated criteria for Reporting Qualitative research Checklist; Table S1: Interview questions for parent interviews; Table S2: Interview questions for service provider interviews. Table S3: Summary of co-design workshops conducted with parents and service providers (Phase 2); Figure S2: Study online registration and consent forms.

## Abbreviations

The following abbreviations are used in this manuscript.

PaRK	Parenting Resilient Kids program
PiP Kids	Partners in Parenting Kids program
PiP Kids-Autism	Partners in Parenting Kids-Autism program

## References

[B1-ejihpe-16-00071] Adams D. (2022). Child and parental mental health as correlates of school non-attendance and school refusal in children on the Autism Spectrum. Journal of Autism and Developmental Disorders.

[B2-ejihpe-16-00071] American Psychiatric Association (2022). Neurodevelopmental disorders: Autism spectrum disorder. Diagnostic and statistical manual of mental disorders.

[B3-ejihpe-16-00071] Australian Bureau of Statistics (2024). Autism in Australia, 2022: Key findings in plain language.

[B4-ejihpe-16-00071] Barter C., Renold E. (2000). “I wanna tell you a story”: Exploring the application of vignettes in qualitative research with children and young people. International Journal of Social Research Methodology.

[B5-ejihpe-16-00071] Bate P., Robert G. (2006). Experience-based design: From redesigning the system around the patient to co-designing services with the patient. BMJ Quality & Safety.

[B6-ejihpe-16-00071] Bearss K., Johnson C., Handen B., Smith T., Scahill L. (2013). A pilot study of parent training in young children with autism spectrum disorders and disruptive behavior. Journal of Autism and Developmental Disorders.

[B7-ejihpe-16-00071] Bearss K., Johnson C., Smith T., Lecavalier L., Swiezy N., Aman M., McAdam D. B., Butter E., Stillitano C., Minshawi N. (2015). Effect of parent training vs parent education on behavioral problems in children with autism spectrum disorder: A randomized clinical trial. JAMA.

[B8-ejihpe-16-00071] Braun V., Clarke V. (2021). To saturate or not to saturate? Questioning data saturation as a useful concept for thematic analysis and sample-size rationales. Qualitative Research in Sport, Exercise and Health.

[B9-ejihpe-16-00071] Braun V., Clarke V., Hayfield N., Davey L., Jenkinson E., Bager-Charleson S., McBeath A. (2022). Doing Reflexive Thematic Analysis. Supporting research in counselling and psychotherapy: Qualitative, quantitative, and mixed methods research.

[B10-ejihpe-16-00071] Buchanan-Pascall S., Gray K. M., Gordon M., Melvin G. A. (2018). Systematic review and meta-analysis of parent group interventions for primary school children aged 4–12 years with externalizing and/or internalizing problems. Child Psychiatry and Human Development.

[B11-ejihpe-16-00071] Cao A., Wu L., Melvin G., Cardamone-Breen M., Broomfield G., Seguin J., Salvaris C., Xie J., Basur D., Bartindale T., McNaney R., Olivier P., Yap M. B. H. (2025). Empowering parents of adolescents at elevated risk of suicide: Co-designing an adaptation to a coach-assisted, digital parenting intervention. European Journal of Investigation in Health, Psychology and Education.

[B12-ejihpe-16-00071] Catalano D., Holloway L., Mpofum E. (2018). Mental health interventions for parent carers of children with autistic spectrum disorder: Practice guidelines from a Critical Interpretive Synthesis (CIS) systematic review. International Journal of Environmental Research and Public Health.

[B13-ejihpe-16-00071] Chan V., Albaum C. S., Khanlou N., Westra H., Weiss J. A. (2025). Parent involvement in mental health treatment for autistic children: A grounded theory-informed qualitative analysis. Child Psychiatry & Human Development.

[B14-ejihpe-16-00071] Cheng W. M., Smith T. B., Butler M., Taylor T. M., Clayton D. (2023). Effects of parent-implemented interventions on outcomes of children with autism: A meta-analysis. Journal of Autism and Developmental Disorders.

[B15-ejihpe-16-00071] Chinsen A., Berg A., Nielsen S., Trewella K., Cronin T. J., Pace C. C., Pang K. C., Tollit M. A. (2025). Co-design methodologies to develop mental health interventions with young people: A systematic review. The Lancet Child & Adolescent Health.

[B16-ejihpe-16-00071] Clark A. (2004). The mosaic approach and research with young children. The reality of research with children and young people.

[B17-ejihpe-16-00071] Clark A. (2010). Transforming children’s spaces: Children’s and adults’ participation in designing learning environments.

[B18-ejihpe-16-00071] DeFilippis M. (2018). Depression in children and adolescents with Autism Spectrum Disorder. Children.

[B19-ejihpe-16-00071] den Houting J. (2021). Participatory and inclusive autism research practice guides.

[B20-ejihpe-16-00071] Department of Health and Aged Care (2024). Intellectual disability health capability framework *(Policy, strategy or framework)*.

[B21-ejihpe-16-00071] Escoffery C., Lebow-Skelley E., Udelson H., Böing E. A., Wood R., Fernandez M. E., Mullen P. D. (2019). A scoping study of frameworks for adapting public health evidence-based interventions. Translational Behavioral Medicine.

[B22-ejihpe-16-00071] Fernando L. M. N., Sim W. H., Jorm A. F., Rapee R., Lawrence K. A., Yap M. B. H. (2018). Parenting Resilient Kids (PaRK), an online parenting program to prevent anxiety and drpression problems in primary school-aged children: Study protocol for a randomised controlled trial. Trials.

[B23-ejihpe-16-00071] Fernández Cerero J., Montenegro Rueda M., López Meneses E. (2024). The impact of parental involvement on the educational development of students with Autism Spectrum Disorder. Children.

[B24-ejihpe-16-00071] Flower R. L., Benn R., Bury S., Camin M., Muggleton J., Richardson E. K., Bulluss E. K., Calabria B., Curran A., Giugni M., Gottliebsen V., Hodges H., Lawrence J., Leung V., Levy-Knoll R., Miklosi K., Mitchelson M., Nuske A., Waldie C., Jellett R. (2025). Defining neurodiversity affirming psychology practice for autistic adults: A Delphi study integrating psychologist and client perspectives. Autism in Adulthood.

[B25-ejihpe-16-00071] Fu C., Chien W. T., Zhang Y., Lam K. K. (2025). Strength-based capacity-building interventions to promote adolescents’ mental health: A systematic review and meta-analysis. European Child & Adolescent Psychiatry.

[B26-ejihpe-16-00071] Fulgoni C. M. F., Melvin G. A., Jorm A. F., Lawrence K. A., Yap M. B. H. (2019). The Therapist-assisted Online Parenting Strategies (TOPS) program for parents of adolescents with clinical anxiety or depression: Development and feasibility pilot. Internet Interventions.

[B27-ejihpe-16-00071] Gotham K., Brunwasser S. M., Lord C. (2015). Depressive and anxiety symptom trajectories from school age through young adulthood in samples with autism spectrum disorder and developmental delay. Journal of the American Academy of Child & Adolescent Psychiatry.

[B28-ejihpe-16-00071] Izuno-Garcia A. K., McNeel M. M., Fein R. H. (2023). Neurodiversity in promoting the well-being of children on the autism spectrum. Child Care in Practice.

[B29-ejihpe-16-00071] Khor S. P. H., Fulgoni C. M., Lewis D., Melvin G. A., Jorm A. F., Lawrence K., Bei B., Yap M. B. H. (2022). Short-term outcomes of the therapist-assisted online parenting strategies intervention for parents of adolescents treated for anxiety and/or depression: A single-arm double-baseline trial. Australian & New Zealand Journal of Psychiatry.

[B30-ejihpe-16-00071] Kienhuis M., Avdagic E. (2021). Parental self-care and self-compassion.

[B31-ejihpe-16-00071] Kim J. A., Szatmari P., Bryson S. E., Streiner D. L., Wilson F. J. (2000). The prevalence of anxiety and mood problems among children with autism and asperger syndrome. Autism.

[B32-ejihpe-16-00071] Maenner M. J. (2020). Prevalence of autism spectrum disorder among children aged 8 years—Autism and developmental disabilities monitoring network, 11 Sites, United States, 2016. MMWR. Surveillance Summaries.

[B33-ejihpe-16-00071] Makino A., Hartman L., King G., Wong P. Y., Penner M. (2021). Parent experiences of autism spectrum disorder diagnosis: A scoping review. Review Journal of Autism and Developmental Disorders.

[B34-ejihpe-16-00071] Malterud K., Siersma V. D., Guassora A. D. (2016). Sample size in qualitative interview studies: Guided by information power. Qualitative Health Research.

[B35-ejihpe-16-00071] Melchior M., van der Waerden J. (2016). Parental influences on children’s mental health: The bad and the good sides of it. European Child & Adolescent Psychiatry.

[B36-ejihpe-16-00071] Morgan A. J., Chittleborough P., Jorm A. F. (2016). Self-help strategies for sub-threshold anxiety: A Delphi consensus study to find messages suitable for population-wide promotion. Journal of Affective Disorders.

[B37-ejihpe-16-00071] National Commission on Safety and Quality in Healthcare (2021). Partnering with consumers standard|Australian commission on safety and quality in health care.

[B38-ejihpe-16-00071] O’Nions E., Happé F., Evers K., Boonen H., Noens I. (2018). How do parents manage irritability, challenging behaviour, non-compliance and anxiety in children with autism spectrum disorders? A meta-synthesis. Journal of Autism and Developmental Disorders.

[B39-ejihpe-16-00071] Oppenheim D., Mottes-Peleg M., Hamburger L., Slonim M., Maccabi Y., Yirmiya N. (2025). The social skills of autistic boys in preschool: The contributions of their dyadic and triadic interactions with their parents. Journal of Child Psychology and Psychiatry.

[B40-ejihpe-16-00071] Paley J. (2025). Qualitative research methods and phenomenology. Encyclopedia of phenomenology.

[B41-ejihpe-16-00071] Parenting Strategies Program (2014). How to reduce your child’s risk of depression and clinical anxiety: Strategies for parents of primary-school aged children.

[B42-ejihpe-16-00071] Pearson H., Myall M., Darlington A.-S., Gibson F. (2025). The approach and application of analysing inductive and deductive datasets: A worked example using reflexive thematic analysis. Qualitative Research in Psychology.

[B43-ejihpe-16-00071] Pellicano E., Dinsmore A., Charman T. (2014). Views on researcher-community engagement in Autism research in the United Kingdom: A mixed-methods study. PLoS ONE.

[B44-ejihpe-16-00071] Perihan C., Burke M., Bowman-Perrott L., Bicer A., Gallup J., Thompson J., Sallese M. (2020). Effects of cognitive behavioral therapy for reducing anxiety in children with high functioning ASD: A systematic review and meta-analysis. Journal of Autism and Developmental Disorders.

[B45-ejihpe-16-00071] Pezzimenti F., Han G. T., Vasa R. A., Gotham K. (2019). Depression in youth with autism spectrum disorder. Child and Adolescent Psychiatric Clinics of North America.

[B46-ejihpe-16-00071] Roche L., Adams D., Clark M. (2021). Research priorities of the autism community: A systematic review of key stakeholder perspectives. Autism.

[B47-ejihpe-16-00071] Rogers S. J., Estes A., Vismara L., Munson J., Zierhut C., Greenson J., Dawson G., Rocha M., Sugar C., Senturk D., Whelan F., Talbott M. (2019). Enhancing low-intensity coaching in parent implemented early start denver model intervention for early autism: A randomized comparison treatment trial. Journal of Autism and Developmental Disorders.

[B48-ejihpe-16-00071] Salomone E., Maurizio Arduino G. (2017). Parental attitudes to a telehealth parent coaching intervention for autism spectrum disorder. Journal of Telemedicine and Telecare.

[B49-ejihpe-16-00071] Sanders E. B.-N., Stappers P. J. (2008). Co-creation and the new landscapes of design. CoDesign.

[B50-ejihpe-16-00071] Sáez-Suanes G. P., Álvarez-Couto M. (2022). Factors associated with quality of life in adults with autism spectrum disorder: A systematic review. Review Journal of Autism and Developmental Disorders.

[B51-ejihpe-16-00071] Schaaf R. C., Toth-Cohen S., Johnson S. L., Outten G., Benevides T. W. (2011). The everyday routines of families of children with autism: Examining the impact of sensory processing difficulties on the family. Autism.

[B52-ejihpe-16-00071] Shochet I. M., Saggers B. R., Carrington S. B., Orr J. A., Wurfl A. M., Duncan B. M. (2019). A strength-focused parenting intervention may be a valuable augmentation to a depression prevention focus for adolescents with autism. Journal of Autism and Developmental Disorders.

[B53-ejihpe-16-00071] Siller M., Sigman M. (2002). The behaviors of parents of children with autism predict the subsequent development of their children’s communication. Journal of Autism and Developmental Disorders.

[B54-ejihpe-16-00071] Sim W. H., Fernando L. M. N., Jorm A. F., Rapee R. M., Lawrence K. A., Mackinnon A. J., Yap M. B. H. (2020). A tailored online intervention to improve parenting risk and protective factors for child anxiety and depression: Medium-term findings from a randomized controlled trial. Journal of Affective Disorders.

[B55-ejihpe-16-00071] Sim W. H., Jorm A. F., Lawrence K. A., Yap M. B. H. (2019). Development and evaluation of the Parenting to Reduce Child Anxiety and Depression Scale (PaRCADS): Assessment of parental concordance with guidelines for the prevention of child anxiety and depression. PeerJ.

[B56-ejihpe-16-00071] Sim W. H., Jorm A. F., Yap M. B. H. (2022). The role of parent engagement in a web-based preventive parenting intervention for child mental health in predicting parenting, parent and child outcomes. International Journal of Environmental Research and Public Health.

[B57-ejihpe-16-00071] Skilling K., Stylianides G. J. (2020). Using vignettes in educational research: A framework for vignette construction. International Journal of Research & Method in Education.

[B58-ejihpe-16-00071] Slattery P., Saeri A. K., Bragge P. (2020). Research co-design in health: A rapid overview of reviews. Health Research Policy and Systems.

[B59-ejihpe-16-00071] Smout A., Melvin G., Cardamone-Breen M., Jorm A., Xie J., Bartindale T., Olivier P., Seguin J., Wu L., Yap M. B. H. (2025). A coach-assisted, online parenting programme to support parents of adolescents who refuse school: Evidence of acceptability and feasibility. BJPsych Open.

[B60-ejihpe-16-00071] Tellegen C. L., Sanders M. R. (2014). A randomized controlled trial evaluating a brief parenting program with children with autism spectrum disorders. Journal of Consulting and Clinical Psychology.

[B61-ejihpe-16-00071] Tong A., Sainsbury P., Craig J. (2007). Consolidated criteria for reporting qualitative research (COREQ): A 32-item checklist for interviews and focus groups. International Journal for Quality in Health Care.

[B62-ejihpe-16-00071] Tonge B., Brereton A., Kiomall M., Mackinnon A., Rinehart N. J. (2014). A randomised group comparison controlled trial of ‘preschoolers with autism’: A parent education and skills training intervention for young children with autistic disorder. Autism.

[B63-ejihpe-16-00071] Turner E. H., Mueller C. W. (2025). Therapeutic focus on strengths is associated with improved functioning and higher clinical progress in children’s public mental health care. Community Mental Health Journal.

[B64-ejihpe-16-00071] UK Design Council (2019). Framework for innovation: Design council’s evolved double diamond.

[B65-ejihpe-16-00071] van Manen M. (2016). Researching lived experience: Human science for an action sensitive pedagogy.

[B66-ejihpe-16-00071] van Steensel F. J. A., Bögels S. M., Perrin S. (2011). Anxiety disorders in children and adolescents with autistic spectrum disorders: A meta-analysis. Clinical Child and Family Psychology Review.

[B67-ejihpe-16-00071] Vargas C., Whelan J., Brimblecombe J., Allender S. (2022). Co-creation, co-design, co-production for public health—A perspective on definition and distinctions. Public Health Research & Practice.

[B68-ejihpe-16-00071] Waters L., Sun J. (2016). Can a brief strength-based parenting intervention boost self-efficacy and positive emotions in parents?. International Journal of Applied Positive Psychology.

[B69-ejihpe-16-00071] White J., Williams P. J., McGarry S., Black M. H. (2025). “Let them show what they can do”: Parents’ views on strengths-based approaches for autistic high school students. Educational Review.

[B70-ejihpe-16-00071] White S. W., Oswald D., Ollendick T., Scahill L. (2009). Anxiety in children and adolescents with autism spectrum disorders. Clinical Psychology Review.

[B71-ejihpe-16-00071] Williams G., Sears L., Allard A. (2006). Parent perceptions of efficacy for strategies used to facilitate sleep in children with autism. Journal of Developmental and Physical Disabilities.

[B72-ejihpe-16-00071] Yap M. B. H., Fowler M., Reavley N., Jorm A. F. (2015). Parenting strategies for reducing the risk of childhood depression and anxiety disorders: A Delphi consensus study. Journal of Affective Disorders.

[B73-ejihpe-16-00071] Yap M. B. H., Jorm A. F. (2015). Parental factors associated with childhood anxiety, depression, and internalizing problems: A systematic review and meta-analysis. Journal of Affective Disorders.

[B74-ejihpe-16-00071] Yap M. B. H., Lawrence K. A., Rapee R. M., Cardamone-Breen M. C., Green J., Jorm A. F. (2017). Partners in parenting: A multi-level web-based approach to support parents in prevention and early intervention for adolescent depression and anxiety. JMIR Mental Health.

[B75-ejihpe-16-00071] Yap M. B. H., Morgan A. J., Cairns K., Jorm A. F., Hetrick S. E., Merry S. (2016). Parents in prevention: A meta-analysis of randomized controlled trials of parenting interventions to prevent internalizing problems in children from birth to age 18. Clinical Psychology Review.

[B76-ejihpe-16-00071] Zaboski B. A., Storch E. A. (2018). Comorbid autism spectrum disorder and anxiety disorders: A brief review. Future Neurology.

[B77-ejihpe-16-00071] Zeidan J., Fombonne E., Scorah J., Ibrahim A., Durkin M. S., Saxena S., Yusuf A., Shih A., Elsabbagh M. (2022). Global prevalence of autism: A systematic review update. Autism Research.

