# Supplementary material for: Co-Designing a Digital Coach-Supported Parenting Program for Internalising Problems in Autistic Children
Source: Eur J Investig Health Psychol Educ. 2026 May 21;16(5):71. doi: 10.3390/ejihpe16050071 (PMC13205567; doi:10.3390/ejihpe16050071)
Supplement: Supplementary file 1 [file ejihpe-16-00071-s001.zip › ejihpe-4214326-supplementary.pdf]

Supplementary Materials

Figure S1

COREQ (COnsolidated criteria for REporting Qualitative research) Checklist

Topic	Item No.	Guide Questions/Description	Reported on Page No.
**Domain 1: Research team and reflexivity**			
Personal characteristics			
Interviewer/facilitator	1	Which author/s conducted the interview or focus group?	9-10
Credentials	2	What were the researcher's credentials? E.g. PhD, MD	9-10
Occupation	3	What was their occupation at the time of the study?	9-10
Gender	4	Was the researcher male or female?	9
Experience and training	5	What experience or training did the researcher have?	9-10
Relationship with participants			
Relationship established	6	Was a relationship established prior to study commencement?	8-10
Participant knowledge of the interviewer	7	What did the participants know about the researcher? e.g. personal goals, reasons for doing the research	8
Interviewer characteristics	8	What characteristics were reported about the interviewer/facilitator? e.g. Bias, assumptions, reasons and interests in the research topic	9-10
**Domain 2: Study design**			
Theoretical framework			
Methodological orientation and Theory	9	What methodological orientation was stated to underpin the study? e.g. grounded theory, discourse analysis, ethnography, phenomenology, content analysis	10
Participant selection			
Sampling	10	How were participants selected? e.g. purposive, convenience, consecutive, snowball	8-10
Method of approach	11	How were participants approached? e.g. face-to-face, telephone, mail, email	8-10
Sample size	12	How many participants were in the study?	8-9
Non-participation	13	How many people refused to participate or dropped out? Reasons?	19
Setting			
Setting of data collection	14	Where was the data collected? e.g. home, clinic, workplace	10, 18-19
Presence of nonparticipants	15	Was anyone else present besides the participants and researchers?	N/A
Description of sample	16	What are the important characteristics of the sample? e.g. demographic data, date	8-9
Data collection			
Interview guide	17	Were questions, prompts, guides provided by the authors? Was it pilot tested?	10, 18-20
Repeat interviews	18	Were repeat inter views carried out? If yes, how many?	N/A

Audio/visual recording	19	Did the research use audio or visual recording to collect the data?	10, 19
Field notes	20	Were field notes made during and/or after the interview or focus group?	10
Duration	21	What was the duration of the inter views or focus group?	10, 19
Data saturation	22	Was data saturation discussed?	11
Transcripts returned	23	Were transcripts returned to participants for comment and/or correction?	10
**Topic**	**Item No.**	**Guide Questions/Description**	**Reported on Page No.**
**Domain 3: analysis and findings**			
Data analysis			
Number of data coders	24	How many data coders coded the data?	11, 20-21
Description of the coding tree	25	Did authors provide a description of the coding tree?	N/A
Derivation of themes	26	Were themes identified in advance or derived from the data?	11, 20-21
Software	27	What software, if applicable, was used to manage the data?	11
Participant checking	28	Did participants provide feedback on the findings?	N/A
Reporting			
Quotations presented	29	Were participant quotations presented to illustrate the themes/findings? Was each quotation identified? e.g. participant number	12-18, 21-23
Data and findings consistent	30	Was there consistency between the data presented and the findings?	-
Clarity of major themes	31	Were major themes clearly presented in the findings?	12-18, 21-23
Clarity of minor themes	32	Is there a description of diverse cases or discussion of minor themes?	12-18, 21-23

Developed from: Tong A, Sainsbury P, Craig J. Consolidated criteria for reporting qualitative research (COREQ): a 32-item checklist for interviews and focus groups. *International Journal for Quality in Health Care*. 2007. Volume 19, Number 6: pp. 349 – 357

Table S1
Interview questions for Parent interviews

Part 1: Understanding parent's experience

1. Tell me about your world.
   Prompts:
   • What is a good day for you?
   • What is a bad day for you?

2. What is the behaviour/difficulty that worries you the most about your child(ren)?

3. Have there been any joys of being a parent to your autistic child?
   Prompts:
   • What has increased the joys of parenting [your autistic child]? What do you enjoy about being a parent to [an autistic child]?

4. What do you find most challenging about being a parent to [an autistic child]? Is there a specific age and stage you particularly like/ excel in/find trickier?

5. Which are the things/factors/people/services/values/places/experiences/qualities that make parenting easier or harder?

Part 2: Parenting programs

6. What are your experiences of using technology (such as on the computer/tablet/ smartphone) to find information or support on parenting your child?
   Prompt:
   • Can you give me some examples of some apps that you enjoy using?

7. Which parenting or family-based programs or interventions have you participated in/been referred to by others?

8. [If relevant] When you registered for the PaRK program, were you looking for something specific to help your children? Can you tell me a bit about your experience of the program?

9. What are the skills, behaviours or outcomes which you want to see improvements in after investing time and effort to complete a parenting program?

10. To what extent do you think current parenting programs work well to support parents of children with autism?
   Prompts:
   • What aspects of (online) parenting programs do you enjoy the most?
   • What aspects do you find most challenging? Which aspects need adaptations?
   • (on personalisation/tailoring) In your opinion, what needs to be done to ensure that a parenting program or intervention is helpful to individual parents/families

11. What ways do you think technology could make it more/less difficult to get help?

12. In what ways could program developers/clinicians better support parents like you?

Table S2

Interview questions for Service Provider interviews

Part 1: Professional background

1. What does a typical day engaging with parents in your job look like?

2. What kinds of supports are parents/families seeking when they come to see you?

Part 2: Understanding the needs of parents/families of children with autism

3. What aspects of parenting an autistic child do you think your clients find most challenging?

4. Why do you think some parents do (or do not) seek parenting help when their child has mental health problems like co-occurring anxiety and depression problems?

Part 3: Views and experiences with technology and (online) parenting programs

5. Which programs or interventions have you referred parents to?

   Prompt:

   • How about parenting programs or services delivered remotely (i.e. online or technology-assisted)?

6. In what ways do you think current parenting programs work well to support parents of autistic children?

7. What aspects of (online) parenting programs do you think parents might enjoy the most?

8. What aspects do you think they might find most challenging? Which aspects need adaptations?

9. (on personalisation/tailoring) In your opinion, what needs to be done to ensure that a parenting program or intervention is helpful to individual parents/families?

10. Why do you think some parents choose not to engage with parenting programs?

11. In what ways could program developers/clinicians better support parents?

Part 4: Intervention outcomes of importance

12. In your opinion, how could one tell if a parent or child has benefited from an intervention? What would you look for as indicators of progress and outcomes?

Table S3

Summary of co-design workshops conducted with parents and service providers (Phase 2)

Work shop	Date	Participant Group	Attendees	Planned Workshop Activities
1	April 2022	Parent	P3, P4	Opening discussion<br>Three Wishes activity<br>Magic Carpet activity
2	May 2022	Parent	P1, P2	
3	May 2022	Service Provider	SP1	Opening discussion<br>Magic Carpet activity<br>Case Vignette
4	June 2022	Service Provider	SP2, SP4	Magic Carpet activity<br>Case Vignette
5	August 2022	Parent	P3, P4	Magic Carpet activity<br>Towers, Winds, and Storms
6	October 2022	Service Provider	SP5	Towers, Winds, and Storms<br>Case Vignette
7	June 2023	Parent	P1, P2, P3, P4	Collaboratively workshop prototype module ‘Minding the self and finding your tribe’, focused on body’s response to stress, parent self-care and finding like-minded supportive people.
8	October 2023	Parent	P1, P3	Collaboratively workshop prototype module ‘Autism: The Fundamentals’, focused on neurodiversity, neurotypes, and parenting journey.

Figure S2

Study online registration and consent forms

ONLINE REGISTRATION AND CONSENT FORM (Parent)

Empowering Parents to Co-create and Evaluate an Online Parenting Program to Reduce Internalising Problems in Children on the Autism Spectrum — A*SPaRK

Project Number: 30478

Thank you for your interest in this project. We are inviting a) parents whose child is currently attending a mainstream primary school, and b) parents who have an older child who has finished school (mainstream or special school) to participate in this project.
If you have yet to chat with the research team or would like more information, please contact Wan Sim at wan.sim@monash.edu

If you have spoken with the research team and have decided to participate, please answer the questions below and click "Agree".

**Name**	
**Contact number**	
**Email address**	
**Preferred day and time for first online meeting (based on your availability over the next 3 weeks)**	1. __________<br>2. __________<br>3. __________<br>4. __________

**Which of the following collaborative and video-conferencing tools do you currently use?**	☐ Basecamp<br>☐ Coviu<br>☐ Dropbox<br>☐ Google Workspace/ G Suite (Docs/Sheets/Drive/Jamboard)<br>☐ GoToMeeting<br>☐ Jira<br>☐ Microsoft (MS) Teams<br>☐ Miro<br>☐ MURAL<br>☐ Slack<br>☐ Webex<br>☐ Zoom<br>☐ Others
**How confident do you feel about your knowledge and skills in parenting your child with autism?**	☐ Not at all confident<br>☐ A little confident<br>☐ Quite confident<br>☐ Very confident
**How important is learning new information and ways of doing things to you?**	☐ Not at all important<br>☐ A little important<br>☐ Quite important<br>☐ Very important
**How important is working alongside researchers to design and run studies to you?**	☐ Not at all important<br>☐ A little important<br>☐ Quite important<br>☐ Very important
**Is there anything that we need to know so that we could make it easier for you to engage with the project activities in the next 2- 3months?**	☐ Yes. Please share a little more ______________________.<br>☐ No.

By clicking the "Agree" button below, I confirm that I have read and understood the explanation statement <insert copy to view/download> and in particular:

- I understand that my involvement in this research will include completing a three-part series of online activities, and up to two surveys. I understand that the online activities are not a treatment program for my child or family;
- I understand that sessions with the researchers and/or other participants will be audio recorded or video recorded to assist with transcription and coding, and that these files will be saved securely and accessed only by members of the research team;
- I understand that transcriptions of the video/audio-recordings will be conducted using a reputable transcription software;
- I have had any questions answered to my satisfaction;

- I understand the risks involved;
- I understand that there may be no direct benefit to me from my participation in this research and that I am free to take part in any other interventions or treatments during my participation in this project;
- I agree to the use of online collaborative tools (e.g., Google suite, MS Team, Zoom) for the duration of my participation;
- I agree to maintain the confidentiality of the information shared by or discussed with other participants and researchers;
- I agree not to share any content/materials developed from this project without the prior permission of other participants and researchers;
- I understand that my participation in this research is voluntary; and that my decision to participate or not, will in no way impact upon my or my child's relationship with Monash University or its staff;
- I understand that if I have any additional questions, I can contact the research team;
- I understand that I am free to withdraw at any time, without explanation or penalty. However, once the data has been transcribed or processed for prototyping, the data will be anonymised and therefore it will not be possible to withdraw my data after this time;
- I understand that my name and other personal information that could identify me or my family will be removed or de-identified in reports, publications or presentations resulting from this research;
- I understand that I can contact the Executive Officer, Monash University Human Research Ethics Committee (MUHREC) on 03 9905 2052 (or email: muhrec@monash.edu) if I have any concerns about the ethical conduct of the project quoting project number 30478.

☐ **I agree to participate in this project.**

ONLINE REGISTRATION AND CONSENT FORM (Service Provider)

Empowering Parents to Co-create and Evaluate an Online Parenting Program to Reduce Internalising Problems in Children on the Autism Spectrum — A*SPaRK

Project Number: 30478

Thank you for your interest in this project. We are inviting professionals and service providers who support parents and families with a child with autism (5-18 years).
If you have yet to chat with the research team or would like more information, please contact Wan Sim at wan.sim@monash.edu

If you have spoken with the research team and have decided to participate, please answer the questions below and click "Agree".

**Name**	
**Contact number**	
**Email address**	
**Preferred day and time for first online meeting (based on your availability for the next 3 weeks)**	1. ____________<br>2. ____________<br>3. ____________<br>4. ____________

**Which of the following collaborative and video-conferencing tools do you currently use?**	☐ Basecamp<br>☐ Coviu<br>☐ Dropbox<br>☐ Google Workspace/ G Suite (Docs/Sheets/Drive/Jamboard)<br>☐ GoToMeeting<br>☐ Jira<br>☐ Microsoft (MS) Teams<br>☐ Miro<br>☐ MURAL<br>☐ Slack<br>☐ Webex<br>☐ Zoom<br>☐ Others
**How confident do you feel about your knowledge and skills in supporting parents of children with autism?**	☐ Not at all confident<br>☐ A little confident<br>☐ Quite confident<br>☐ Very confident
**How important is learning new information and ways of doing things to you?**	☐ Not at all important<br>☐ A little important<br>☐ Quite important<br>☐ Very important
**How important is working alongside researchers to design and run studies to you?**	☐ Not at all important<br>☐ A little important<br>☐ Quite important<br>☐ Very important
**Is there anything that we need to know so that we could make it easier for you to engage with the project activities in the next 2- 3months?**	☐ Yes. Please share a little more ____________________.<br>☐ No.

By clicking the "Agree" button below, I confirm that I have read and understood the explanation statement <insert copy to view/download> and in particular:

- I understand that my involvement in this research will include completing a three-part series of online activities, and up to two surveys. I understand that the online activities are not a training program for me and are not a treatment program for my clients;
- I understand that sessions with the researchers and/or other participants will be audio recorded or video recorded to assist with transcription and coding, and that these files will be saved securely and accessed only by members of the research team;

- I understand that transcriptions of video/audio-recordings will be conducted using a reputable transcription software;
- I have had any questions answered to my satisfaction;
- I understand the risks involved and that there may be no direct benefit to me from my participation in this research;
- I understand that my participation in this research is voluntary; and that my decision to participate or not, will in no way impact upon my relationship with Monash University or its staff;
- I agree to the use of online collaborative tools (e.g., Google suite, MS Team, Zoom) for the duration of my participation;
- I agree to maintain the confidentiality of the information shared by or discussed with other participants and researchers;
- I agree not to share any content/materials developed from this project without the prior permission of other participants and researchers;
- I understand that if I have any additional questions, I can contact the research team;
- I understand that I am free to withdraw at any time, without explanation or penalty. However, once the data has been transcribed or processed for prototyping, the data will be anonymised and therefore it will not be possible to withdraw my data after this time;
- I understand that my name and other personal information that could identify me will be removed or de-identified in reports, publications or presentations resulting from this research;
- I understand that I can contact the Executive Officer, Monash University Human Research Ethics Committee (MUHREC) on 03 9905 2052 (or email: muhrec@monash.edu) if I have any concerns about the ethical conduct of the project quoting project number 30478.

☐ **I agree to participate in this project.**
